# Implementation, intervention, and downstream costs for implementation of a multidisciplinary complex pain clinic in the Veterans Health Administration

**DOI:** 10.1111/1475-6773.14345

**Published:** 2024-07-02

**Authors:** Sarah I. Daniels, Shayna Cave, Todd H. Wagner, Taryn A. Perez, Sara N. Edmond, William C. Becker, Amanda M. Midboe

**Affiliations:** ^1^ Center for Innovation to Implementation (Ci2i) VA Palo Alto Health Care System Menlo Park California USA; ^2^ Health Economics and Research Center Center for Policy Evaluation Veterans Affairs Palo Alto Health Care System Palo Alto California USA; ^3^ Department of Surgery Stanford University Palo Alto California USA; ^4^ Pain Research, Informatics, Multimorbidities and Education (PRIME) Center for Innovation VA Connecticut Healthcare System West Haven Connecticut USA; ^5^ Yale School of Medicine New Haven Connecticut USA; ^6^ Department of Public Health Sciences, Division of Health Policy and Management University of California Davis—School of Medicine Davis California USA

**Keywords:** chronic pain, costs and cost analysis, economic evaluation, healthcare costs, implementation science

## Abstract

**Objective:**

To determine the budget impact of implementing multidisciplinary complex pain clinics (MCPCs) for Veterans Health Administration (VA) patients living with complex chronic pain and substance use disorder comorbidities who are on risky opioid regimens.

**Data Sources and Study Setting:**

We measured implementation costs for three MCPCs over 2 years using micro‐costing methods. Intervention and downstream costs were obtained from the VA Managerial Cost Accounting System from 2 years prior to 2 years after opening of MCPCs.

**Study Design:**

Staff at the three VA sites implementing MCPCs were supported by Implementation Facilitation. The intervention cohort was patients at MCPC sites who received treatment based on their history of chronic pain and risky opioid use. Intervention costs and downstream costs were estimated with a quasi‐experimental study design using a propensity score‐weighted difference‐in‐difference approach. The healthcare utilization costs of treated patients were compared with a control group having clinically similar characteristics and undergoing the standard route of care at neighboring VA medical centers. Cancer and hospice patients were excluded.

**Data Collection/Extraction Methods:**

Activity‐based costing data acquired from MCPC sites were used to estimate implementation costs. Intervention and downstream costs were extracted from VA administrative data.

**Principal Findings:**

Average Implementation Facilitation costs ranged from $380 to $640 per month for each site. Upon opening of three MCPCs, average intervention costs per patient were significantly higher than the control group at two intervention sites. Downstream costs were significantly higher at only one of three intervention sites. Site‐level differences were due to variation in inpatient costs, with some confounding likely due to the COVID‐19 pandemic. This evidence suggests that necessary start‐up investments are required to initiate MCPCs, with allocations of funds needed for implementation, intervention, and downstream costs.

**Conclusions:**

Incorporating implementation, intervention, and downstream costs in this evaluation provides a thorough budget impact analysis, which decision‐makers may use when considering whether to expand effective programming.

**What is known on this topic**

- Opioid‐related morbidity and mortality affect over 2 million Americans per year and have extraordinarily high economic and societal costs.
- Healthcare systems urgently need treatment models that address patients living with chronic pain who are prescribed opioids and have substance use disorders.
- Healthcare decision‐makers need cost information to determine how and when to implement multidisciplinary clinics for those living with complex chronic pain.

**What this study adds**

- This study evaluates the initial start‐up costs of implementing a multidisciplinary pain clinic for patients living with complex chronic pain and taking risky opioids at three distinct VA hospitals, relying on Implementation Facilitation.
- Implementation of multidisciplinary complex pain clinics require fairly little funding for implementation costs and more substantial funding for intervention and downstream costs to meet the needs of serving patients living with complex chronic pain.
- Relatively similar budget impacts were observed at the facility‐level across all three disparate VA sites, demonstrating robust estimates when presenting this clinic to decision‐makers at new sites.

## 1 INTRODUCTION

Opioid use disorder (OUD) and opioid overdose deaths in the United States were estimated to cost $1.02 trillion in 2017, which includes healthcare‐related costs and losses in quality of life.^1^ Over‐prescribing of opioids for pain contributed to the opioid overdose epidemic,^2^ which has been further exacerbated by both the COVID‐19 pandemic^3^, ^4^, ^5^ and an increase in supply of illicitly‐manufactured fentanyl.^6^, ^7^ Treatment approaches that focus on populations at risk of opioid overdose, including those living with chronic pain, OUD, and other mental health comorbidities, are urgently needed.

Guideline‐concordant treatment for patients living with chronic pain and substance use disorder requires both pharmacological and nonpharmacological modalities (NPMs).^8^, ^9^ Studies have shown improvements in pain and function^10^, ^11^, ^12^ and reduced opioid use^13^, ^14^ when patients engage with NPMs. The Veterans Health Administration (VA) has been innovative in increasing access to NPMs for chronic pain as well as pharmacological treatments such as buprenorphine.^8^, ^15^ VA/DoD jointly report on buprenorphine in their clinical practice guidelines as a safe, efficacious, and well‐tolerated medication as compared with full agonists.^16^ At the VA, buprenorphine can be favored by veteran patients and providers (e.g., over methadone)^17^ and it significantly reduces healthcare costs as compared with methadone treatment.^18^ In the broader population, buprenorphine is cost‐effective as compared to no addiction treatment.^19^, ^20^ Similarly, there are cost‐effectiveness data to support the use of NPM strategies, including combined exercise and psychological treatments as well as cognitive‐behavioral therapy and mindfulness‐based stress reduction for managing chronic pain.^21^, ^22^

Barriers to implementing buprenorphine can include inadequate provider training and experience^23^, ^24^ staff and resource limitations,^23^, ^24^ and stigma around OUD,^17^ while barriers to NPMs include provider misconceptions about administering NPMs to their patients.^25^, ^26^ The opioid reassessment clinic model was developed to focus on serving patients with chronic pain on long‐term opioid therapy, many of whom have complex comorbidities such as OUD.^27^, ^28^, ^29^ The multidisciplinary team model evaluated in this study is based on the opioid reassessment clinic model but has a broader mission to use multimodal treatment those living with chronic pain, and as such, we refer to it as the multidisciplinary complex pain clinic (MCPC).

The primary aim of MCPCs is to safely and effectively manage a patient's complex chronic pain, including engagement with NPMs, thus improving both patient functioning and quality of life.^28^ The MCPC relies on the multidisciplinary expertise of clinicians in pain management, addiction, psychiatry, pharmacy, clinical/health psychology, and nursing to best support patients. Once risky opioid regimens are addressed and improved pain management approaches applied in the MCPC, patients return to their primary care provider with support from MCPC clinicians as needed. Economic uncertainties regarding the budgetary impact of the MCPC for such a high‐need/high‐cost population may cause some medical center leadership to be reluctant to adopt this evidence‐based model.^30^ The objective of this study was to estimate the budgetary impact of implementing the MCPC across multiple sites so that decision‐makers can be more informed about the initial funding required.

## 2 METHODS

### 2.1 Overview

Each MCPC was staffed by a multidisciplinary team that included an internist with pain management expertise, addiction psychiatrist, nurse care manager, and a clinical/health psychologist.^28^ The two main functions of the MCPC are: (1) to help stabilize complex chronic pain patients who are at risk of experiencing adverse effects due to their current opioid regimen, and (2) to support primary care providers' ability to manage these patients over time.

Three VA medical centers implemented an MCPC beginning in August 2020, February 2021, and March 2021; hereafter, we refer to these as intervention sites. A budget impact analysis was conducted by estimating implementation costs, intervention costs, and downstream costs.^31^ We used a short‐term time horizon with the VA's facility‐level perspective following recommendations.^32^, ^33^ Implementation costs were measured at the three MCPC sites, where we focused on labor costs and excluded fixed costs associated with space given the short‐term time horizon. To measure incremental intervention and downstream costs, we conducted a difference‐in‐differences (DiD) analysis by comparing patients treated at intervention sites to clinically similar patients at control sites.

Administrative data from the VA's Corporate Data Warehouse were used to identify MCPC‐treated and control patients; VA's Managerial Cost Accounting (MCA) system was used to estimate costs.^34^, ^35^, ^36^, ^37^ Veterans' demographic and clinical characteristics (medical histories including previous diagnoses, prescriptions, and inpatient/outpatient visits) were pulled from the Corporate Data Warehouse. All patient‐level encounters at VA facilities were recorded in the MCA extracts using an activity‐based cost accounting method and annual costs were adjusted for inflation to 2023 dollars using the Consumer Price Index for all Urban Consumers.^38^ We used primary and secondary stop codes to categorize the types of outpatient care.

### 2.2 Site selection and target population

The intervention group was defined as patients who attended an MCPC due to a medical history of chronic pain and risky opioid regimens, usually presenting with comorbid substance use disorders and/or other psychiatric disorders. Patients were referred to the MCPC by their primary care provider (details of referral in Figure S1a–c). There was rolling entry into the treatment cohort, and patients were followed in the MCPC until deemed stable and could be returned to their primary care provider's panel. At one intervention site (Site 2), roughly 30% (*n* = 105) of MCPC patients had pre‐exposure to a consult‐and‐recommendation only pain clinic, and therefore this subset of patients was removed from this cost analyses so as not to contaminate the pre‐intervention period.

We conducted a quasi‐experimental implementation study design and selected our control sites based on contextual factors. To limit confounders, including the impact of geographic variation, control patients were chosen from VA medical centers in the same Veterans Integrated Service Network as the MCPCs. These 18 networks are geographic regions of the United States that support regional financing in VA. We identified multiple candidate control sites for each intervention site and narrowed our selection after obtaining data about the sites from our national pain management program partner. For one intervention site, we included all candidate control sites (*n* = 7) as all had existing pain clinics. For each of the remaining two intervention sites, two candidate control sites were excluded because there was no staffing for pain management at these locations; thus, the total number of included control sites was *n* = 5 (71%) and *n* = 4 (66%). Control patients were selected based on medical histories that were similar to the intervention group (as described in Figure S2a–c), such as their diagnoses (described in Table S1) and prescription use. Specifically, this includes pain diagnoses as defined by International Classification of Diseases codes,^39^ diagnoses of OUD or opioid overdose, and prescriptions of long‐term opioid therapy proximal to the time of pain diagnosis (opioids listed in Table S2) or prescriptions of medications for OUD (medications listed in Table S3). Veterans with cancer or in hospice were excluded. Pseudo‐enrollment dates were chosen at random for each control patient. If patients died, all costs after their date of death were set to missing, thereby excluding them from the denominator.

### 2.3 Implementation costs

#### 2.3.1 Implementation strategies

Implementation Facilitation is a suite of implementation activities^40^ to increase the uptake of evidence‐based practices (EBPs),^41^, ^42^ including in primary care and other clinical settings.^43^ Implementation Facilitation was used to support the implementation of the MCPCs, including relying on local internal facilitation teams and an external facilitation team.^44^, ^45^ Internal facilitation teams for the sites included 2‐3 members requiring a general internist and/or a pharmacist and clinic‐level leadership. Internal facilitation responsibilities included hiring or identifying the qualified staff (physicians, clinical/health psychologists, nurses) and setting up the MCPC. The external facilitation team included at least one expert in Implementation Facilitation to lead the meeting and at least one clinical expert. The external team interfaced with the internal team by identifying and assisting in resolutions of barriers to implementation, establishing workflows and referral processes for the MCPC, assessing availability of dashboards for identifying eligible patients, providing monthly audit and feedback, and involving stakeholders and gaining buy‐in from leadership.

#### 2.3.2 Activity‐based costing

We used micro‐costing methods to measure implementation costs.^46^, ^47^ Implementation costs were estimated by recording time dedicated to implementation activities, both general project activities and site‐specific activities as described in Table 1. These data are also used for purposes of developing strategies and/or natural (i.e., unplanned) adaptations to move past obstacles to implement guideline‐concordant pain care. Research Electronic Data Capture‐based activity logs were completed by the external facilitation team to report time spent by each staff member who was associated with the implementation efforts. To estimate costs, total facilitation hours accrued by each staff member was multiplied by an hourly wage. Hourly wages were based on MCA clinical staff wages and Office of Personnel Management wage tables for nonclinicians.^48^ We used wage rates from the Bureau of Labor Statistics (BLS) website to estimate non‐VA costs.^49^ Both Office of Personnel Management and BLS wage data represented salary and wages only. Benefits were estimated as an additional 30%. Accounting was completed for implementation costs at each MCPC site over 2 years; there were no implementation activities at the control sites.

**TABLE 1:** Description of implementation, intervention, and downstream costs from the site‐level perspective.

Cost Type	Definition	Data Source	Outcome measure (denominator)	Specifications
Implementation	Labor‐related “start‐up” costs prior to intervention and continued facilitation concurrent with the intervention	Micro‐costing using surveys that tracked activities	Monthly	Monthly site calls between EF and IF teams EF team engages IF team to support implementation Academic Detailing/Education on unsafe opioid prescribing, identifying patients with chronic pain, and referring to MCPC Problem‐solving based on assessment of implementation barriers and facilitators Developing materials and adding them to a shared library Informing local opinion leaders, leadership, and/or administrators Conducting audit and feedback Discussing incentives in connection with implementation Provide/receive technical assistance
Intervention	MCPC‐related costs	VA administrative data from an activity‐based cost accounting system	Per patient per three‐month interval	Intake visit and follow‐up visits related to the MCPC, including all outpatient nonpharmacological treatments for pain Most stop codes were from existing data‐based metrics identifying guideline‐concordant for pain,^50^ but sites at times suggested modifications during the audit/feedback process, which led to better specification of outpatient care and mental health care. See Table S4.
Downstream	Non‐MCPC‐related outpatient costs (not associated with intervention)	VA administrative data based on an activity‐based cost accounting system	Per patient per three‐month interval	Any outpatient healthcare utilization that is unrelated to the intervention (not included in list of relevant MCPC‐related stop codes) All prescribed pharmacologic formulations are included in downstream costs
	Inpatient related costs	VA administrative data based on an activity‐based cost accounting system	Per patient per three‐month interval	Total inpatient healthcare utilization (reported monthly) for each patient. All inpatient costs were unassociated with the MCPC intervention.

Abbreviations: EF, External Facilitation; IF, Internal Facilitation; MCPC, Multidisciplinary complex pain clinics; VA, Veterans Health Administration.

### 2.4 Intervention costs

The MCPC is designed to increase the use of multimodal pain and substance use disorder care. Intervention costs included the outpatient intake visit to the MCPC (assessment, discussion, treatment planning) and the outpatient follow‐up visits (treatment, prescribing, care coordination). Additional care may include other NPMs such as physical therapy, chiropractic care, and other types of rehabilitation. To acquire relevant costs from the MCA data, we used a combination of date and stop codes to identify the MCPC‐related care for the treated patients and standard route of pain‐related care for the control patients at control sites (Table S4). While the pain clinic stop code was most commonly used to designate MCPC care, we refined a full list of NPM‐related stop codes based on the Opioid Therapy Guideline Adherence Report,^50^ with the addition of two stop codes that were frequently used by MCPC mental health providers for patients in the intervention group.

### 2.5 Downstream costs

We hypothesized that patient healthcare utilization patterns may change for the treated group, as the MCPC events that require in‐person patient visits could lead to patient engagement in additional types of care leading to downstream costs. All nonintervention‐ related healthcare utilizations were considered downstream events.^31^ Downstream costs include: (1) all outpatient care, exclusive of intervention‐related care; (2) a minimal amount of pain‐related care provided outside of the VISN (e.g., a mental healthcare visit far from a patient's usual healthcare center); (3) all pharmacy and laboratory services (these services could not be parsed between intervention and downstream costs); (4) inpatient care costs (as is standard in downstream analyses).

### 2.6 Statistical analysis

We estimated the average monthly implementation costs for each site over the course of the implementation period. Intervention and downstream costs attributable to the MCPC were estimated using a PS‐weighted DiD research design. This DiD was used to estimate the difference in average cost between MCPC sites compared with control sites before and after the start of the MCPC. The DiD generates plausible causal effects if key assumptions are met. The parallel trends assumption implies that the intervention and control groups would maintain similar trajectories in the absence of the MCPC intervention.^51^, ^52^ We assessed the validity of this assumption by running pretests of parallel trends for each model using the event‐study regression included in the DiD statistical package.^53^

To estimate the DiD, we chose the Callaway and Sant'anna (CSDID) estimator over the alternative two‐way fixed effect (TWFE).^53^ CSDID outperforms TWFE and other competing frameworks when confounders may vary over time and sample size is small, although the trade‐off of reduced bias is higher variance .^54^, ^55^ As a sensitivity analysis, we report the results of weighted TWFE results for both intervention and downstream costs as well.

An event study (i.e., dynamic DiD) was used to monitor changes in cost differences preceding and following the opening of the MCPC. While each MCPC site showed some evidence of higher costs and care utilization soon after the opening of the clinic, there was considerable variability in patient utilization and so resulting costs fluctuated widely from month‐to‐month. Therefore, we aggregated the monthly costs into three‐month periods for our analyses and reporting, as similarly done in previous work,^33^ with the time intervals centered on the three‐month period following the opening of the MCPC. Differences in MCPC‐related costs between the intervention and control groups were reported as the static (overall) average treatment effect on the treated (ATT) per patient per three‐month period after the MCPC starting date. The CSDID event studies were used to compare the MCPC treatment group to the controls at three distinct sites in a designated panel study design. This included repeated measures with patient‐level clustering and a doubly robust estimation method with multiplier‐bootstrapped standard errors. The nonzero costs that were above the 99th percentile were trimmed.

The control population meeting our eligibility criteria was substantially larger than the MCPC‐treated group at each site. Given that this could create imbalance when comparing the two groups, we identified a large number of possible control patients, over‐selected for site‐specific variables, and then randomly selected the remaining controls for our analysis as done previously.^56^ Due to inherent differences among VA facilities and target populations, we anticipated demographic and clinical characteristics to differ somewhat between the treated and control groups. To improve the comparability of cases and controls and adjust for observable confounders affecting selection into treatment, we used propensity score (PS) weights,^57^, ^58^, ^59^ including demographic and clinical variables, in the DiD research design. Specifically, we employed inverse probability weights, which are the inverse conditional probability of either receiving the intervention (1/PS) for the treated group or not receiving the intervention (1/(1 − PS)) for the control group.^60^, ^61^ Weights were chosen instead of matching as prior research demonstrates that matching can cause inadvertent imbalance and model dependencies.^62^

The PS model estimated the probability of receiving care from the MCPC. Demographics included sex, race/ethnicity, and age quintiles using the Observational Medical Outcomes Partnership common data model.^63^, ^64^ VA administrative data on race and ethnicity are based on self‐identification, with categorization into major groups observed at each site: non‐Hispanic White (i.e., White), non‐Hispanic Black (i.e., Black), Hispanic ethnicity or other race, and unknown race/ethnicity. Clinical characteristics included previous benzodiazepine prescriptions (listed in Table S5), diagnoses of any substance use disorders and/or common mental health diagnoses, or inpatient stay in the 3 months prior to entry (each described in Table S1 with diagnosis codes in Table S6). Common support was tested using kernel density plots for the scores to examine overlap in PS between the intervention and control groups. Standardized differences were calculated for both weighted and unweighted variables as a metric of imbalance between treated and untreated groups, and we recognized that some imbalance may remain due to small sample sizes in stratified groups.^65^ DiD analyses were performed with and without the PS weights to compare the directionality and magnitude of these outcomes.

## 3 RESULTS

### 3.1 Implementation costs

**FIGURE 1:**
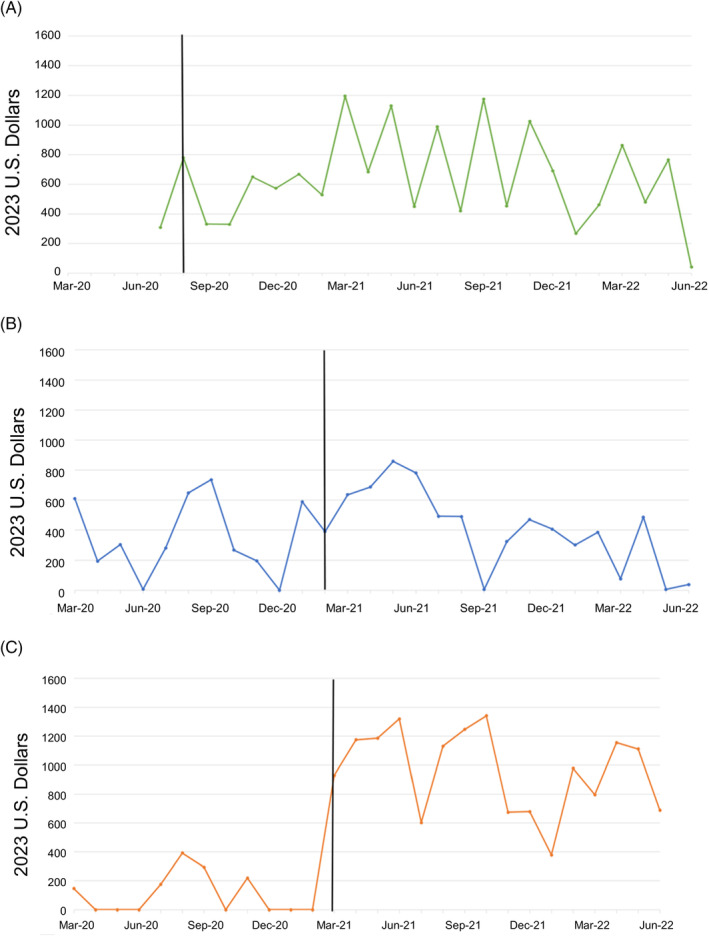
Monthly Implementation Facilitation costs at each site (vertical line indicates first month of implementation). (A) Site 1. (B) Site 2. (C) Site 3.

The site‐level implementation costs average $636 per month for Site 1, $381 per month for Site 2, and $593 per month for Site 3. VA costs are sometimes lower than those in the private sector. As expected, the non‐VA costs, estimated using BLS salaries, were either similar to or slightly higher than VA costs at $710, $414, and $592 per month for Sites 1, 2, and 3, respectively. Figure 1A–C shows the variation in VA costs from month‐to‐month at each site. Differences in cost across sites were related to the frequency of meetings. Higher‐cost months were associated with site‐specific meetings between internal and external implementation teams.

### 3.2 Site‐level differences in patient sample populations

Intervention and downstream costs involved comparison of MCPC patients to control patients at other sites. The three intervention sites had unique clinical and demographics characteristics (Table 2). The distributions of racial/ethnic diversity differed, with Site 1 having greater proportions of self‐identified Black patients than Sites 2 and 3. The proportions of patients having recently diagnosed substance use disorders were higher in Sites 1 and 3 as compared with Site 2, while Sites 2 and 3 had higher proportions of patients with benzodiazepine prescriptions. Standardized mean differences between the treated and control groups showed imbalance in the distribution of some unweighted variables at each site, particularly at Sites 1 and 3; however, most imbalance between groups diminished (below 0.20) after conducting the weighting procedure (Table S7a–c).

**TABLE 2:** Demographic and clinical characteristics of treated and control populations at each site.

	Site 1				Site 2				Site 3			
	Unweighted		Weighted		Unweighted		Weighted		Unweighted		Weighted	
	Case	Control	Case	Control	Case	Control	Case	Control	Case	Control	Case	Control
Total, *N*	322	2898			259	2331			111	999		
Average age	59.5	62.3	61.1	62.1	63.7	64.9	64.6	64.8	66.2	62.7	63.5	62.9
White	63%	60%	61%	61%	84%	83%	84%	84%	92%	89%	92%	90%
Black	33%	35%	35%	35%	2%	7%	6%	6%	2%	4%	2%	4%
Other race/Hisp Ethn	2%	3%	2%	3%	9%	6%	6%	6%	4%	5%	4%	5%
Unknown Race/Ethn	2%	2%	2%	2%	5%	4%	4%	4%	3%	2%	2%	2%
Female	9%	8%	9%	8%	9%	8%	9%	8%	11%	6%	7%	7%
OUD or MOUD	44%	28%	30%	29%	14%	17%	18%	17%	53%	46%	41%	47%
Benzodiazepines	16%	11%	13%	12%	25%	26%	25%	25%	29%	24%	26%	25%
Alcohol use disorder	25%	22%	23%	22%	15%	15%	15%	15%	32%	33%	33%	33%
Stimulant use disorder	15%	10%	11%	10%	3%	4%	4%	4%	8%	17%	15%	16%
Sedative use disorder	3%	2%	3%	2%	2%	3%	3%	3%	5%	6%	6%	6%
Cannabis use disorder	13%	9%	10%	9%	6%	6%	6%	6%	13%	15%	18%	15%
Other substance use disorder	8%	7%	7%	7%	3%	3%	3%	3%	8%	10%	8%	9%
Depression, anxiety, and/or PTSD	77%	66%	70%	68%	61%	63%	64%	63%	77%	72%	72%	73%
Opioid overdose	7%	2%	3%	3%	2%	2%	1%	2%	0%	3%	0%	3%
Inpatient admit prior to enrollment	22%	10%	11%	11%	12%	11%	10%	11%	23%	31%	32%	31%

Abbreviations: Ethn, Ethnicity; Hisp, Hispanic; MOUD, medication for opioid use disorder; OUD, opioid use disorder.

### 3.3 Intervention costs

**FIGURE 2:**
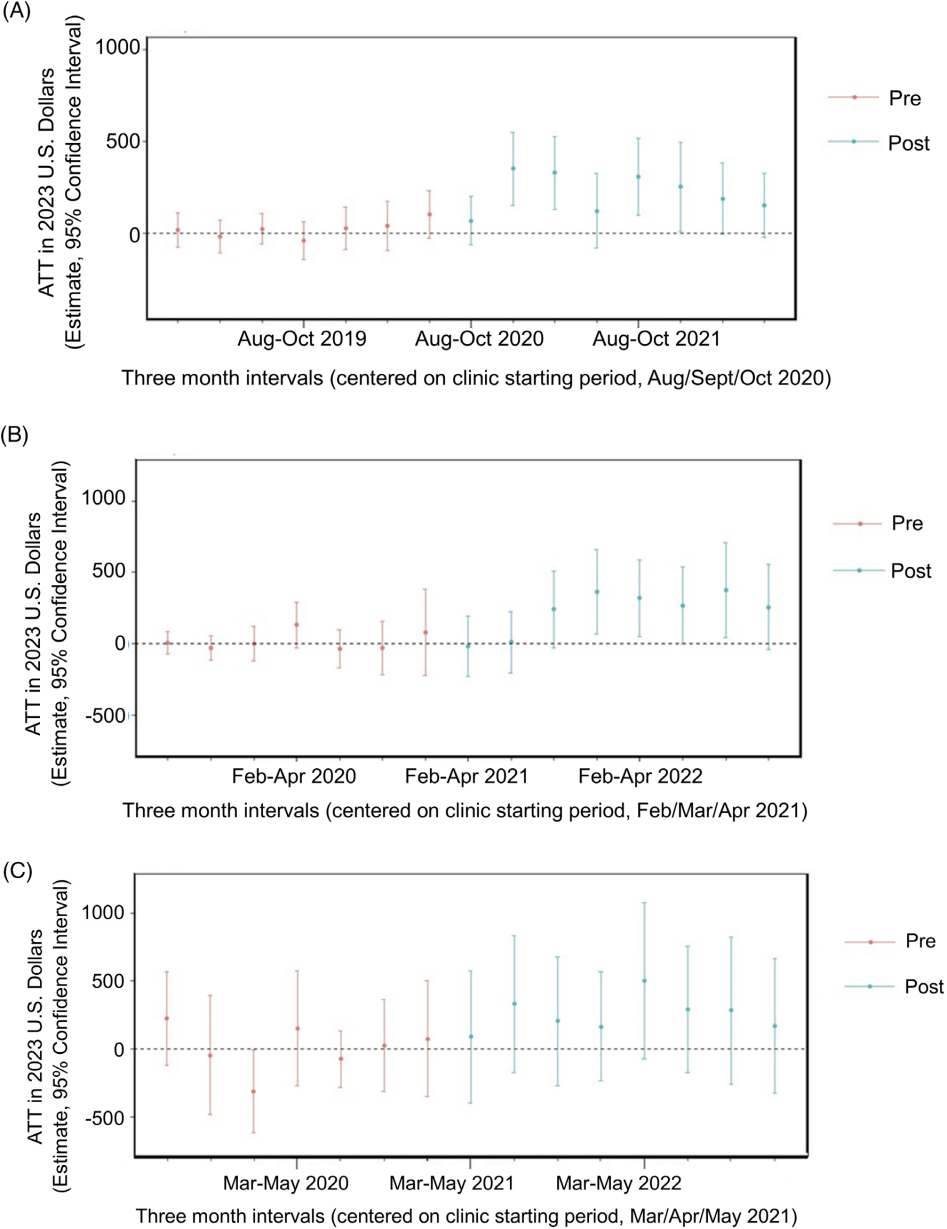
Propensity score‐weighted difference‐in‐difference event studies for site intervention costs. (A) Site 1. Overall average treatment effect of treated (ATT): $220.93 (95% CI 138.17, 303.70) *p* < 0.001. (B) Site 2. Overall average treatment effect of treated (ATT): $271.05 (95% CI 145.46, 396.64) *p* < 0.001. (C) Site 3. Overall average treatment effect of treated (ATT): $253.93 (95% CI −24.33, 532.18) *p* = 0.069.

Figure 2A–C shows the intervention cost results of the PS‐weighted CSDID event study for each site, with the estimated budget impacts per patient corresponding to the cost differences at each three‐month window of exposure to the MCPC. (The baseline and three‐month mean cost comparisons for each site are reported in Table S8 and estimates for each three‐month period of the event study are in Table S9a–c). Site 1 and Site 2 demonstrated significant increases in overall cost per patient in the 2 years post‐implementation as compared with the control group, with reported ATT estimates of $221 (95% CI 138, 304) and $271 (95% CI 145, 397), respectively. Site 3 did not exhibit a significant increase: $254 (95% CI −24, 532). The majority of Site 1 and Site 2's three‐month time intervals are significantly higher than the controls after the first period of the intervention. Unweighted models demonstrate slightly larger effect measures with narrower confidence intervals (data not shown). As a sensitivity analysis, we explored PS‐weighted TWFE models as an alternative to the CSDID framework (Figure S3a–c); TWFE models are more conventionally used for DiD, although they are subject to greater bias. Of note, Site 1 opened their MCPC during the beginning of the pandemic, and the interval prior to the MCPC starting interval coincides with Spring 2020. This time point does show some evidence of a parallel trend violation in the PS‐weighted TWFE model (Figure S3a); however, we found no significant change in the ATT effect measure when shifting the baseline time point back by one interval (data not shown). Pretests for parallel trend assumptions (Table S8) also show a significant violation for Site 3 (due to one time interval), and therefore results should be interpreted with caution.

### 3.4 Downstream costs

**FIGURE 3:**
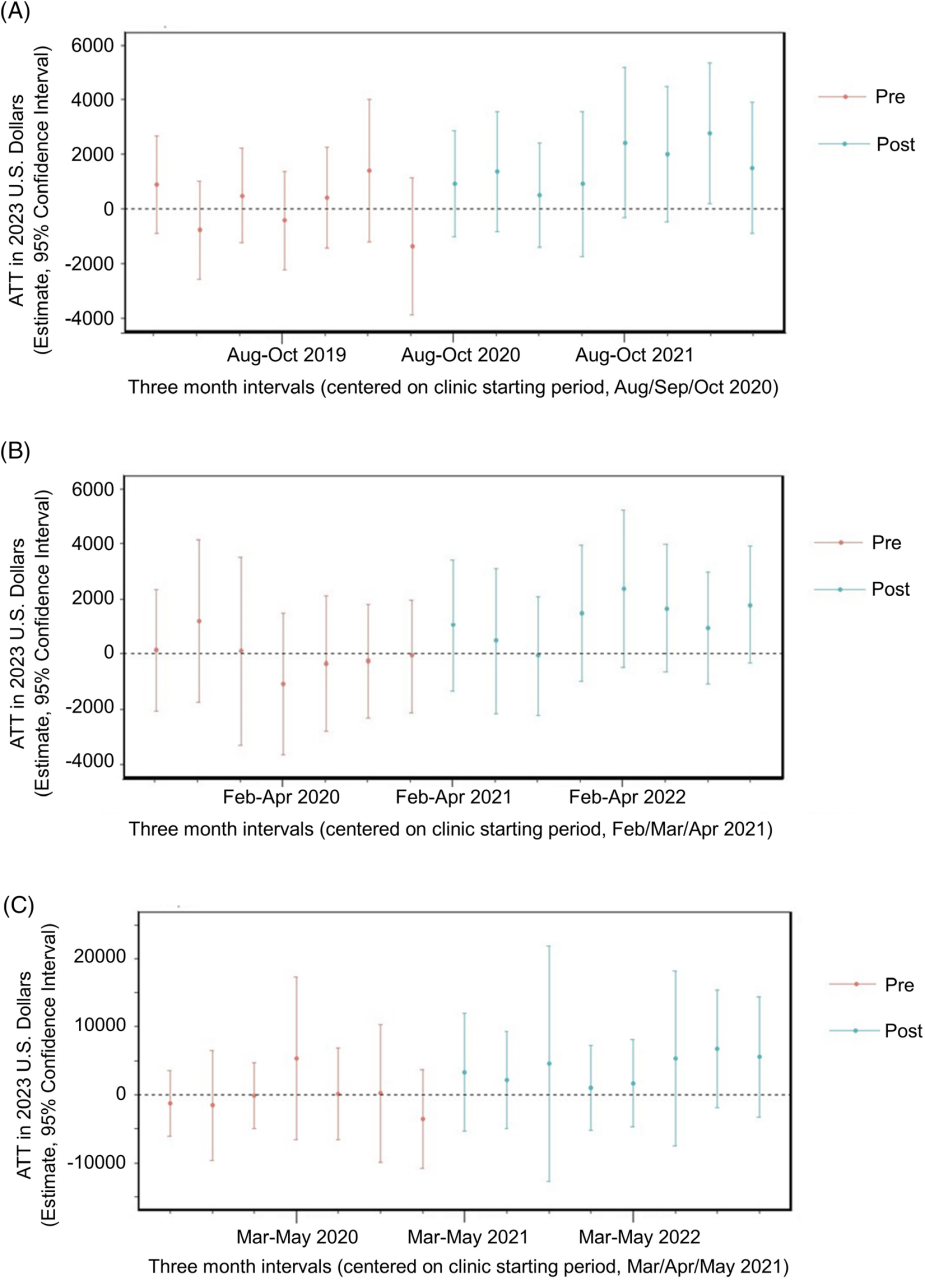
Propensity score‐weighted difference‐in‐difference event studies for site downstream costs. (A) Site 1. Overall average treatment effect of treated (ATT): $1555.93 (95% CI 492.13, 2619.73) *p* < 0.005. (B) Site 2. Overall average treatment effect of treated (ATT): $1217.93 (95% CI −64.62, 2500.48) *p* = 0.054. (C). Site 3. Overall average treatment effect of treated (ATT): $3791.27 (95% CI −690.46, 8273.01) *p* = 0.092.

The overall average downstream costs per patient per three‐month time interval are significantly greater at Site 1 in the PS‐weighted CSDID event study, with an ATT estimate of $1556 (95% CI 492, 2620) (Figure 3A). The measure of effect was similar for Site 2 and Site 3 but not significant, with ATT estimates of $1218 (95% CI −65, 2500) and $3791 (95% CI −690, 8273) (Figure 3B,C). (The baseline and three‐month mean cost comparisons for each site are in Table S8 and corresponding estimates for each time interval are in Table S10a–c). As a reminder, Site 1 opened their MCPC during the pandemic, which accounts for some of the variability observed in the interval prior to the opening of the MCPC, but pretests at all sites show no evidence of violations of the parallel trend assumption (Table S8). The unweighted ATT estimates resulted in less conservative estimates, with larger ATTs and smaller confidence intervals (data not shown).

We conducted several follow‐up analyses to determine whether our results can withstand statistical tests of validity (i.e., sensitivity analyses). PS‐weighted TWFE event‐study results showed similar trends (Figure S4a–c). When examining outpatient costs alone (i.e., removing inpatient costs), significant ATT estimates were observed across all three sites (Figure S5a–c), with smaller effect sizes as compared with the original downstream analysis.

## 4 DISCUSSION

This is the first study to examine the budget impact of implementing a multidisciplinary pain clinic focused on treating patients living with complex chronic pain, who are on risky opioid regimens. We found relatively low *implementation costs* (less than $800) from month‐to‐month when using the evidence‐based Implementation Facilitation strategy.^42^ Joint meetings between internal and external facilitators were the main contributors to cost differentials between sites. These “all‐hands” meetings included discussions on outreach to the target veteran population, patient referral pathways, barriers to implementation, provider education on the MCPCs, and systematization of records for consults and intake MCPC visits. While the implementation costs for these MCPCs have relatively low impact on an overall hospital budget, the variation we observed across facilities can also affect the ability to implement successfully. For example, sites with significant staffing turnover, particularly in specialty care (e.g., addiction, pain management), may not be able to provide staff for an MCPC until those concerns are resolved.

Second, we found that the intervention costs significantly increased at two of the three intervention sites, with an estimated increase of approximately $200 per patient per 90‐day period, and these increased costs appeared to stabilize over time. Of note, the third site had a similar measure of effect, although the treatment group sample size was at least 50% smaller than the two other sites. When extrapolating these intervention costs to other healthcare settings outside of the VA, additional capital costs (e.g., physical space, supplies, technology) may be considered in establishing MCPCs.

Finally, downstream costs were only significantly higher at one intervention site in comparison with its control sites. Of note, there were a small proportion of observed inpatient utilizations as compared with outpatient, and the inpatient costs were substantially larger than outpatient costs. Therefore, the combined outpatient and inpatient variance was naturally inflated, which can lead to reducing statistical power to detect meaningful differences between the treated and control groups. When assessing the overall average effects of the outpatient downstream costs alone, all three sites exhibit significant differences, demonstrating that combining large, infrequent inpatient costs with smaller recurrent outpatient costs can mask important insights in the analyses of the downstream outcome metric.

Budget impact analyses help decision‐makers understand the affordability of adopting a new treatment modality. The strength of this study is that it provides a unified approach to understand the budgetary impact, which is measured by the net effect of implementation, intervention, and downstream costs. This study is novel in its use of micro‐costing for measuring implementation costs, alongside a DiD model to estimate the average intervention and downstream costs. A budget impact analysis should be viewed as complementary to traditional cost‐effectiveness analyses that consider costs over the long run (i.e., 5 or more years). Both analyses address important questions based on different perspectives and time horizons. Budget impact analyses have been conducted for other implementation trials for nonpharmacological treatments for pain.^66^, ^67^ A virtual pain management program recently introduced to patients at a VA site incurred costs over 3 years that are comparable to annual costs of the MCPC intervention (ca. $1000 vs. $800 per patient).^68^ For interventions addressing OUD, the budget impact for external facilitation implementation strategies has been recently reported;^69^ however, intervention and downstream costs have been modeled but not empirically assessed.^70^, ^71^ Simulation methods do provide valuable insights at a high level, but overlook the real‐world expenses that are inherent to implementation, such as costs due to starting‐up a new clinic, early stage learning curves, and unanticipated downstream effects.

Additionally, other cost evaluation studies on multidisciplinary team approaches have addressed patient substance use disorders and serious mental illness in populations similar to the target population for this study (i.e., high‐need, high‐cost patients). A complex care intervention for high‐need, high‐cost patients with a multidisciplinary team was shown to increase outpatient visits while decreasing inpatient admissions and emergency department visits, and as a result did not significantly change overall Medicaid claim costs per patient.^72^ Multidisciplinary clinics embedded in primary care for high‐need, high‐cost patients have demonstrated almost doubling of outpatient costs, but a 16% lower healthcare utilization costs overall due to substantial decreases in inpatient care.^73^ Our sensitivity analyses of outpatient costs showed appreciable increases at all three sites; however, two of three sites no longer showed significant differences between the MCPC‐treated group and the comparator controls when including inpatient utilization in the total downstream costs. As one of the purposes of the MCPC is to reduce burden on primary care and related burnout, future studies may explore the potential of cost savings in mitigating primary care staff turnover. This would especially benefit staff with patient panels largely comprised of patients with complex chronic pain and a history of risky opioid use along with other potential mental health comorbidities.

Although this study provides valuable insight into the cost of implementing multidisciplinary clinics for treating patients living with complex chronic pain, there are some limitations to mention. As the VA is the largest integrated healthcare system in the United States, it has a high level of collaboration within a network of healthcare providers to facilitate a continuum of care, especially for complex patients. Due to the VA's exceptionalism, intervention and downstream costs reported here may not necessarily be representative of costs incurred in nonintegrated healthcare settings. Moreover, both the treated and control sites are in urban metropolitan centers; therefore, additional cost risks (e.g., patient accessibility to site, staff turnover, overburdened providers) would need to be considered when translating these estimates to more rural settings. We spent a considerable amount of time identifying control sites and control patients, while following current recommendations.^74^ While these control sites vary in demographics and clinical characteristics, the target patient sample was selected to best represent those experiencing complex chronic pain and prescribed risky opioid regimens at each site. Additional differences between the case and control sites were accounted for using PS weighting as described; however, these still depend on an assumption that the controls are appropriate. Lastly, it is also important to note that the implementation of MCPCs occurred during the COVID‐19 pandemic, and geographic variation in the transmission of the virus may have confound specific time points in the DiD analysis due to sporadic shifts in overall healthcare utilization across sites.

## 5 CONCLUSION

A comprehensive cost evaluation was conducted to systematically assess implementation, intervention, and downstream budgetary impacts of a multidisciplinary pain clinic for patients living with complex chronic pain. This study addresses some key issues faced in cost evaluations, including accurate reporting of Implementation Facilitation activities and selecting the most representative control group at neighboring sites. Stakeholders and those in leadership at healthcare facilities can better prepare funding allocations when presented with more inclusive cost evaluations at the perspective of the healthcare facility (as opposed to the patient‐level) and when assessed over a two‐year time horizon. This standard of practice can act as a template for other implementation scientists to leverage similar techniques for economic analyses of their hybrid implementation–effectiveness trials, especially when serving complex patient populations.

## Supporting information


**Figure S1.** (a–c) CONSORT Diagrams for identifying treated patients at each of three MCPC sites.

**Figure S2.** (a–c) CONSORT Diagrams for identifying control patients at each of three MCPC sites.

**Table S1‐S10.** Supporting information.

**Supplemental Figure 3a‐c:** Propensity‐score weighted two‐way fixed effects event studies for site‐level intervention costs

**Supplemental Figure 4a‐c:** Propensity‐score weighted two‐way fixed effects event studies for site‐level total downstream costs.

**Supplemental Figure 5a‐c:** Propensity‐score weighted difference‐in‐difference event studies for site‐level outpatient‐only downstream costs.
