# Supplementary material for: Implementation, intervention, and downstream costs for implementation of a multidisciplinary complex pain clinic in the Veterans Health Administration
Source: Health Serv Res. 2024 Jul 2;59(Suppl 2):e14345. doi: 10.1111/1475-6773.14345 (PMC11540574; doi:10.1111/1475-6773.14345)

Supplemental Figures 1a-c. CONSORT Diagrams for identifying treated patients at each of three MCPC Sites

Supplemental Figure 1a. Site 1 workflow

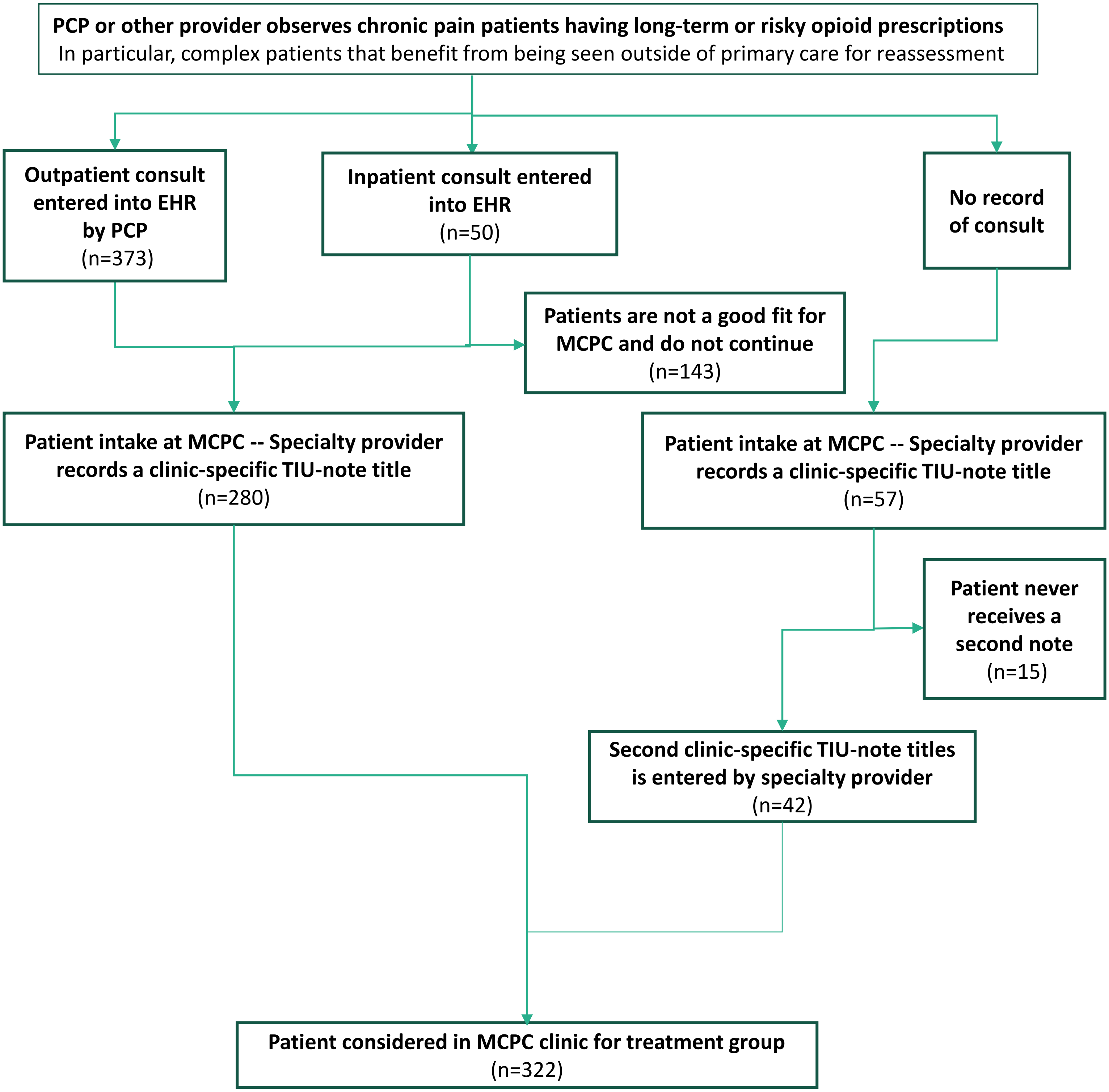

Supplemental Figure 1b. Site 2 Workflow

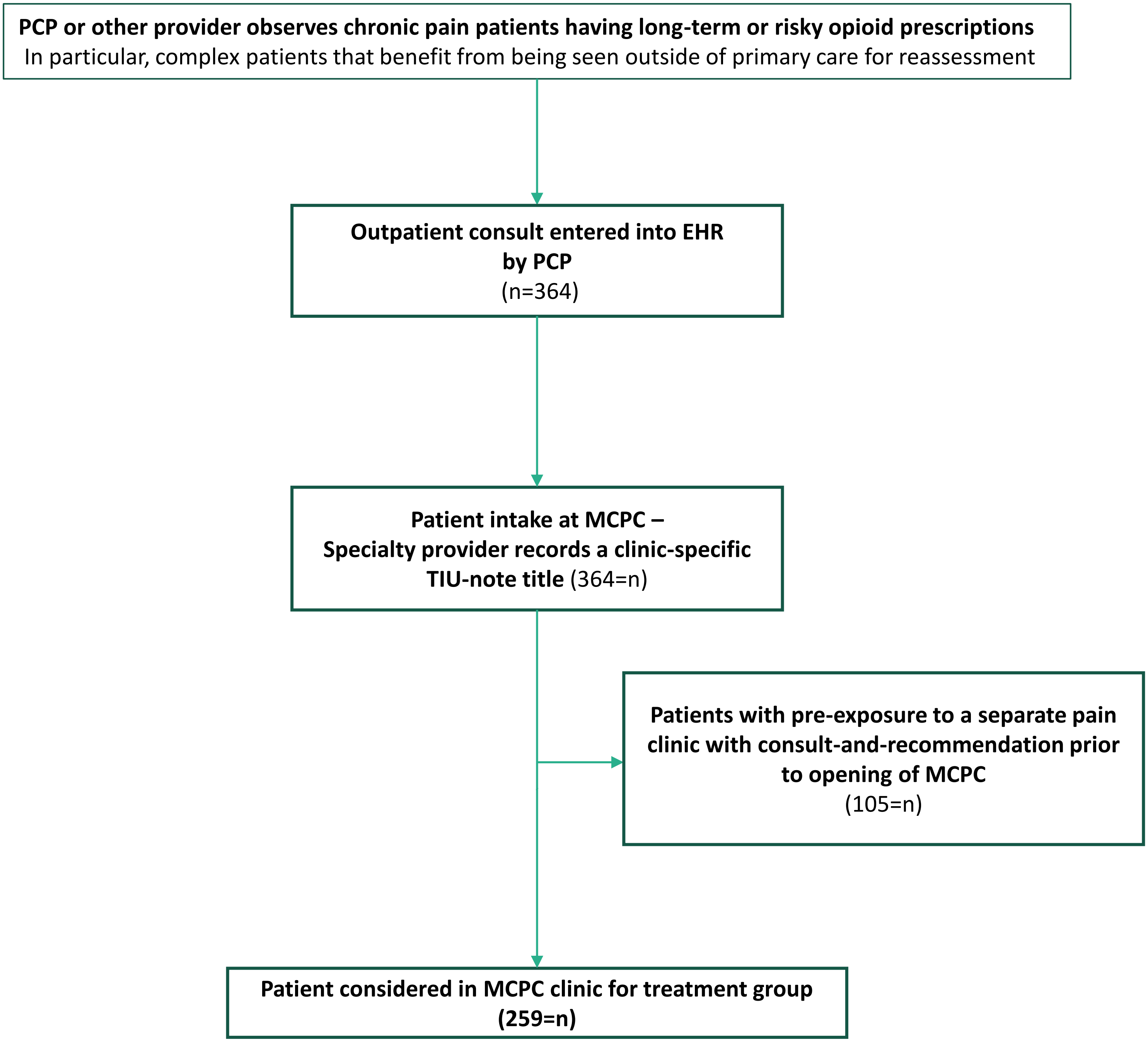

Supplemental Figure 1c. Site 3 Workflow

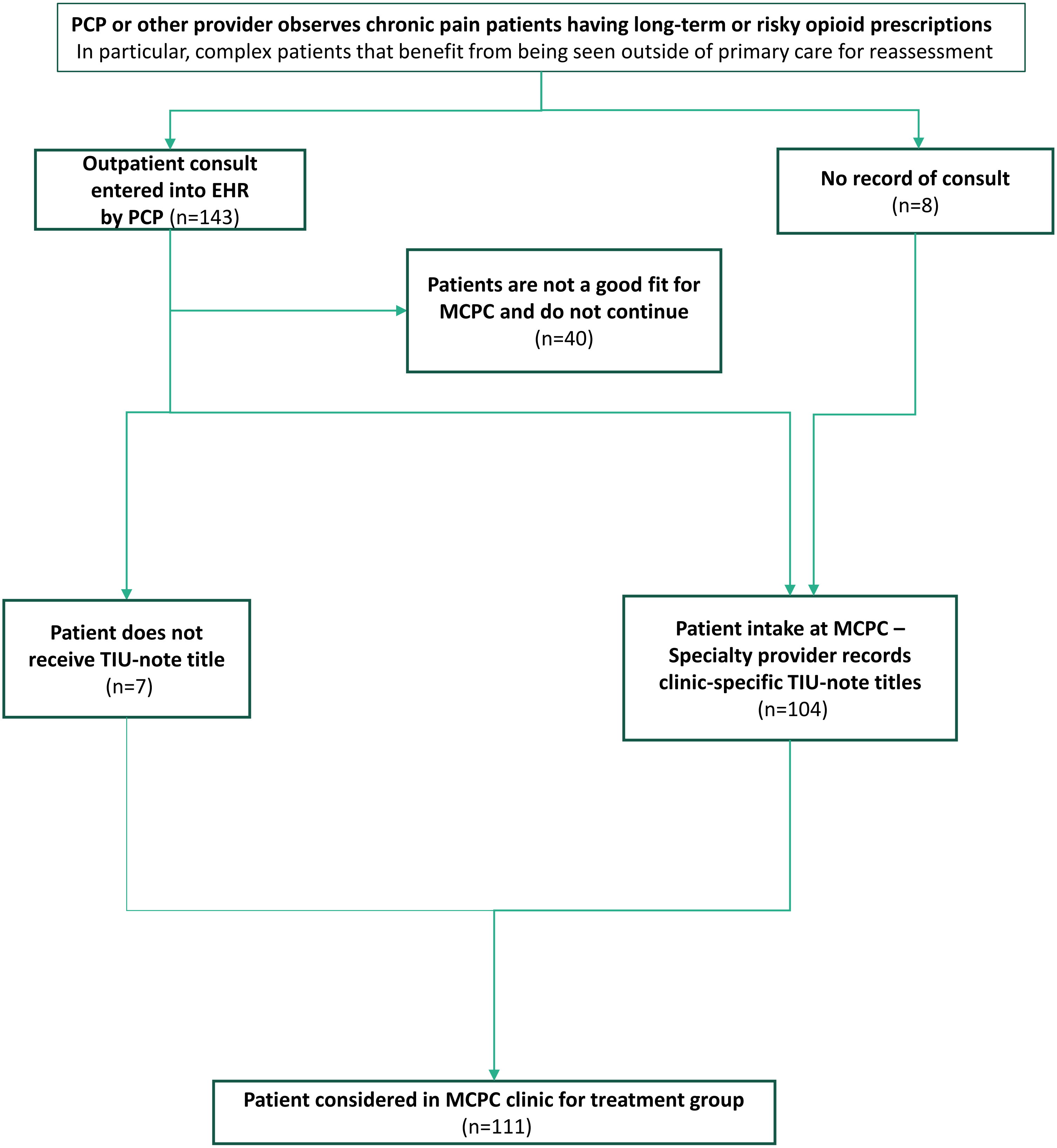

Supplement: Supplementary file 1 — Figure S1. (a–c) CONSORT Diagrams for identifying treated patients at each of three MCPC sites. [file HESR-59-0-s002.pdf]
