# Supplementary material for: Implementation, intervention, and downstream costs for implementation of a multidisciplinary complex pain clinic in the Veterans Health Administration
Source: Health Serv Res. 2024 Jul 2;59(Suppl 2):e14345. doi: 10.1111/1475-6773.14345 (PMC11540574; doi:10.1111/1475-6773.14345)
Supplement: Supplementary file 2 — Figure S2. (a–c) CONSORT Diagrams for identifying control patients at each of three MCPC sites. [file HESR-59-0-s003.pdf]

Supplemental Figures 2a-c. CONSORT Diagrams for identifying control patients at each of three MCPC Sites

Supplemental Figure 2a. Control patient selection from five VA sites within the same VISN as site 1

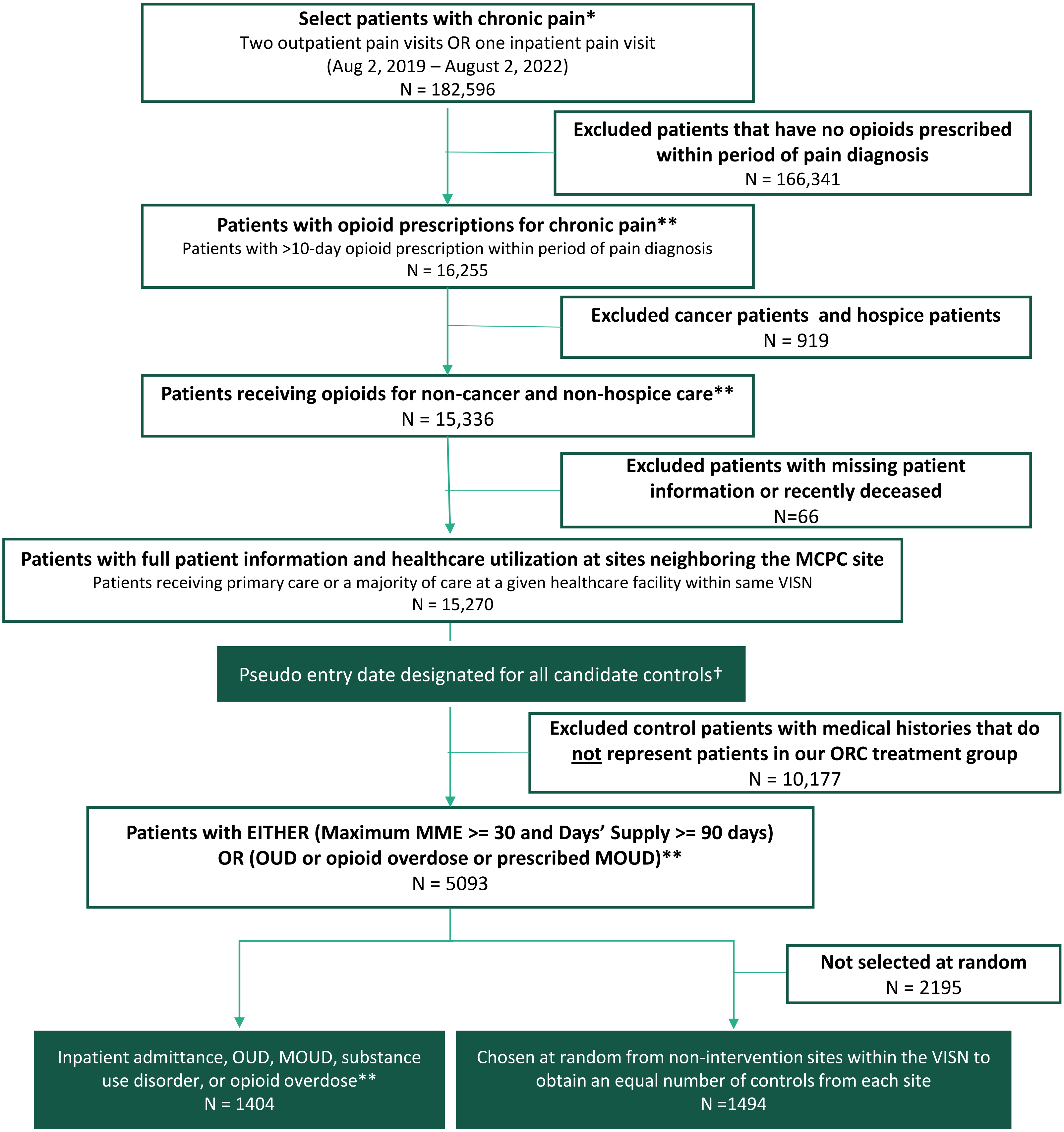

*Chronic pain diagnosis codes were used from the list referenced in Mayhew et al., 2019
**Defined in Supplementary Material
†A pseudo-entry date is defined as a proxy for the entry into the non-intervention arm (to mimic the treated patients' rolling entry into the MCPC treatment group)

Supplemental Figure 2b. Control patient selection from four VA sites within the same VISN as site 2

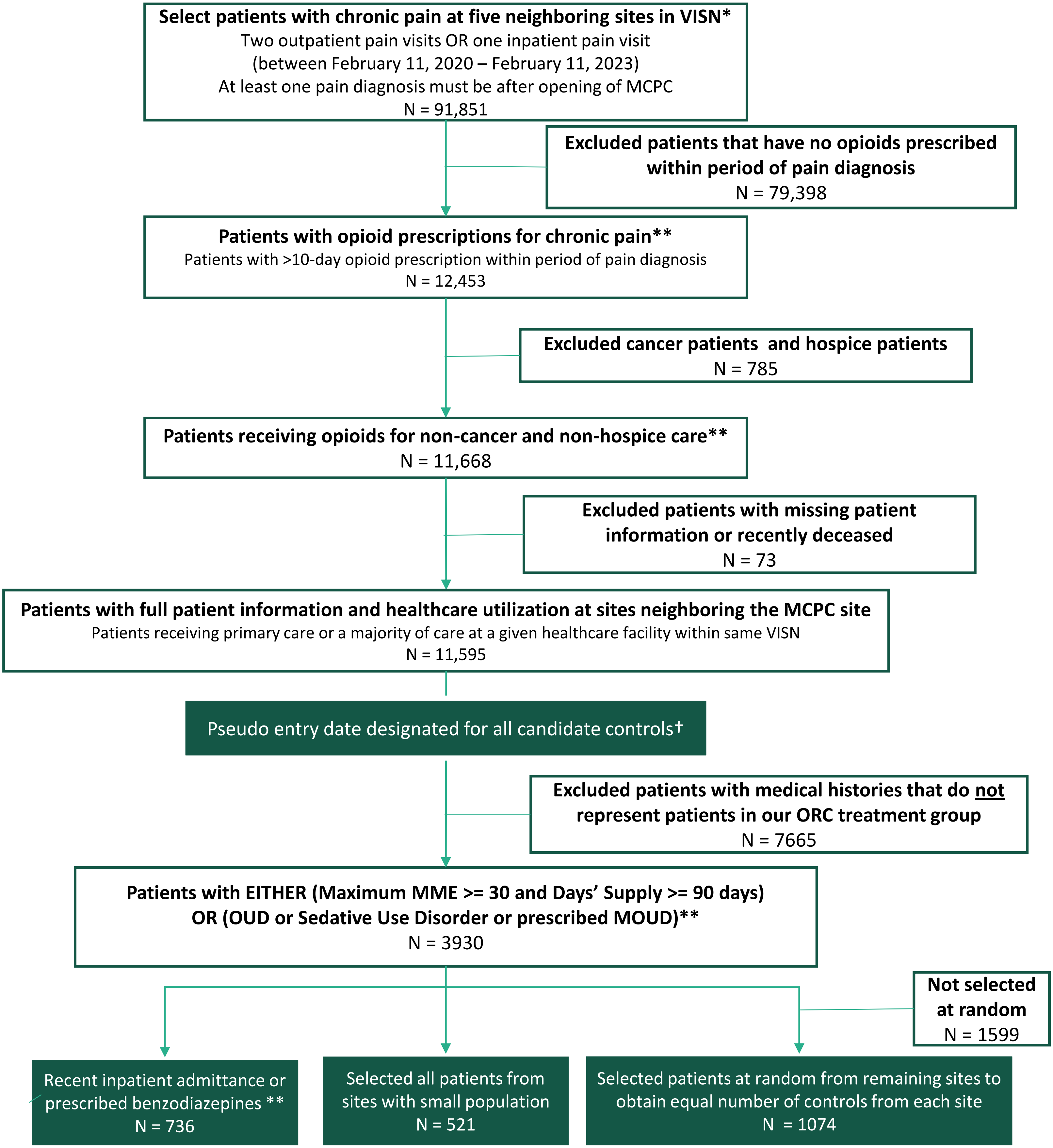

*Chronic pain diagnosis codes were used from the list referenced in Mayhew et al., 2019

**Defined in Supplementary Material

†A pseudo-entry date is defined as a proxy for the entry into the non-intervention arm (to mimic the treated patients' rolling entry into the MCPC treatment group)

Supplemental Figure 2c. Control patient selection from seven VA sites within the same VISN as site 3

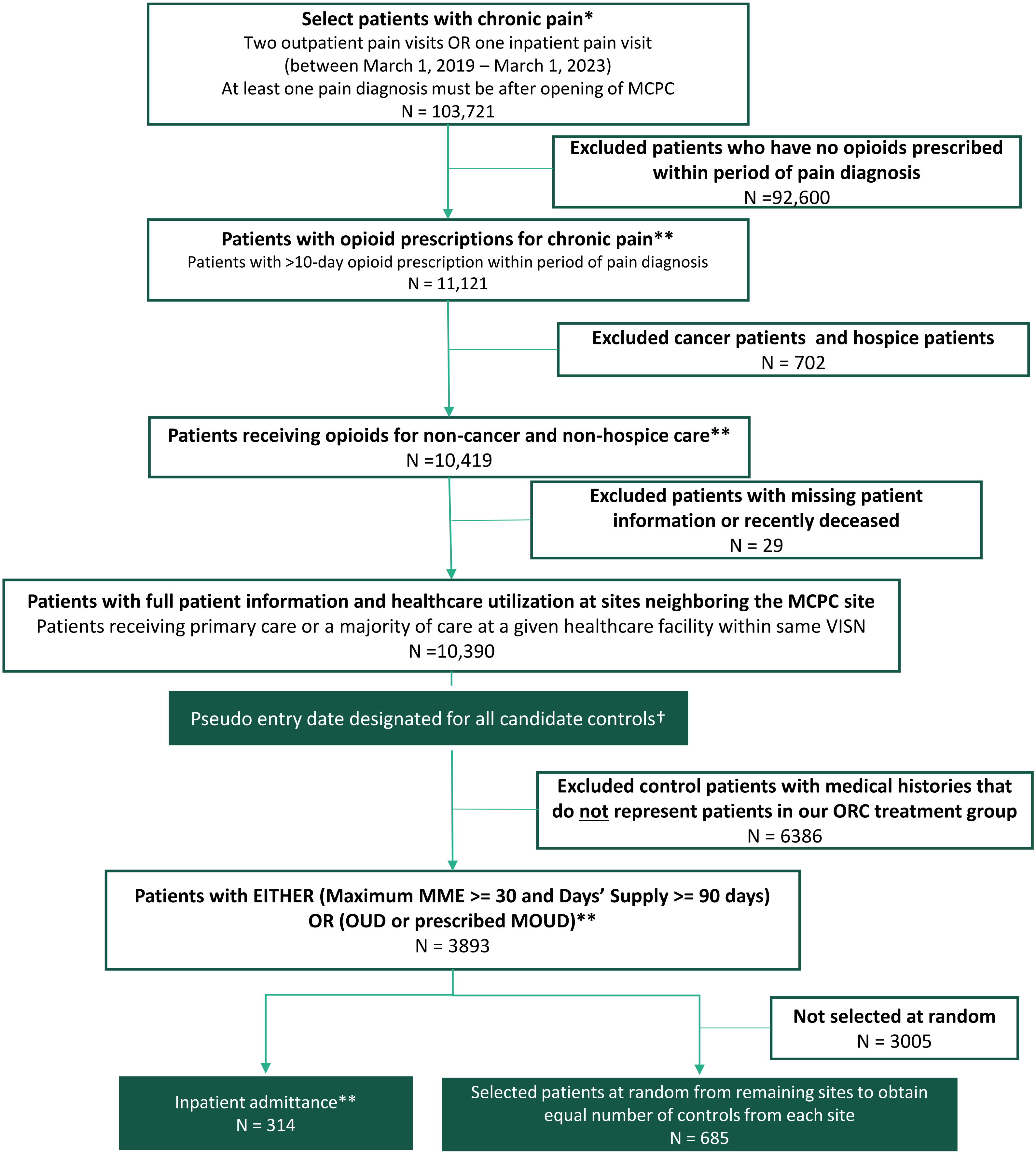

*Chronic pain diagnosis codes were used from the list referenced in Mayhew et al., 2019

**Defined in Supplementary Material

†A pseudo-entry date is defined as a proxy for the entry into the non-intervention arm (to mimic the treated patients' rolling entry into the MCPC treatment group)
