# Supplementary material for: Implementation, intervention, and downstream costs for implementation of a multidisciplinary complex pain clinic in the Veterans Health Administration
Source: Health Serv Res. 2024 Jul 2;59(Suppl 2):e14345. doi: 10.1111/1475-6773.14345 (PMC11540574; doi:10.1111/1475-6773.14345)
Supplement: Supplementary file 3 — Table S1‐S10. Supporting information. [file HESR-59-0-s005.docx]

**Supplemental Table 1. Data definitions for clinical variables and determinants used in identifying the treated and control groups**

| **Variable Name** | **Data Definition** |
| --- | --- |
| Chronic Pain Diagnosis (for control patients) | Two outpatient pain diagnoses or one inpatient discharge using pain ICD^a^ codes (32), with the most recent pain diagnosis reported during the post-intervention period (i.e., after the start date of the clinic) |
| Cancer Diagnosis (for control patients) | Two outpatient or one inpatient cancer diagnosis code AND cancer stop code (94,308,316,330,431,904) within one year prior to pseudo-entry date |
| Pseudo-entry date (for control patients) | To match the treated patients’ rolling entry into the clinic over the course of the two-year observational period, patients in the control group were given a pseudo-entry to mark their beginning of their “entry” period. This date is selected at random within the following range for each unique control patient: [First opioid release date OR first chronic pain diagnosis OR 8/2/2020] TO [Last day opioid supply or last chronic pain diagnosis OR 9/30/2022] |
| Hospice patient (for control patients) | Stop code (351) |
| Opioids Prescriptions (for control patients) | >10 or 14 days supply of opioids prescribed (depending on jurisdiction) during the period of pain diagnosis as defined by satisfying at least one of the following:   - first pain diagnoses was in the window of an opioid prescription release date and last day of supply - last pain diagnoses was in the window of an opioid prescription release date and last day of supply - the opioid prescription release date was between their first pain diagnoses and last pain diagnoses - the last day of opioid supply falls between the first pain diagnoses and the last pain diagnoses   See drug names in Supplemental Table 2 |
| Age, sex, race/ethnicity | Defined using the OMOP^b^ common data model (33,34) |
| Medication for opioid use disorder prescription | Any prescription released two years prior to clinic entry (or pseudo-entry date) and 14-days after entry.  See drug names in Supplemental Table 3 |
| Intervention-related stop codes | See Supplemental Table 4 |
| Benzodiazepine prescriptions | Any prescription released two years prior to clinic entry (or pseudo-entry date) and 14-days after entry.  See drug names in Supplemental Table 5 |
| Inpatient admittance prior to multidisciplinary complex plain clinic | Inpatient stays from 90 -days prior to 7-days after clinic entry or pseudo-entry date |
| Any substance use disorder | Includes stimulant use disorder, sedative use disorder, cannabis use disorder, alcohol use disorder, or other substance use disorders |
| Opioid use disorder, opioid overdose, alcohol use disorder, sedative use disorder, stimulant use disorder, cannabis use disorder, other substance use disorder (hypnotics, inhalants, psychotics), and/or common mental health diagnoses (depression, anxiety, and/or post-traumatic stress disorder) | At least one outpatient ICD^a^ diagnosis code two years prior to entry (or pseudo-entry date) or 14-days after entry into the clinic. See Supplemental Table 6 for ICD^a^ codes used to identify each listed condition |

^a^ ICD: International Classification of Diseases codes; ^b^ OMOP: Observational Medical Outcomes Partnership

**Supplemental Table 2. List of opioids for selection of comparator control group**

| Acetaminophen/Aspirin/Caffeine/Codeine/Salicylamide |
| --- |
| Acetaminophen/Aspirin/Caffeine/Hydrocodone |
| Acetaminophen/Aspirin/Codeine |
| Acetaminophen/Butalbital/Caffeine/Codeine |
| Acetaminophen/Butalbital/Caffeine/Hydrocodone |
| Acetaminophen/Butalbital/Codeine |
| Acetaminophen/Caffeine/Codeine/Salicylamide |
| Acetaminophen/Caffeine/Dihydrocodeine |
| Acetaminophen/Codeine |
| Acetaminophen/Dihydrocodeine/Salicylamide |
| Acetaminophen/Hydrocodone |
| Acetaminophen/Meperidine |
| Acetaminophen/Oxycodone |
| Acetaminophen/Pentazocine |
| Acetaminophen/Propoxyphene |
| Acetaminophen/Tramadol |
| Aluminum Hydroxide/Aspirin/Codeine/Magnesium Hydroxide |
| Aspirin/Butalbital/Caffeine/Codeine |
| Aspirin/Caffeine/Codeine |
| Aspirin/Caffeine/Dihydrocodeine |
| Aspirin/Caffeine/Dihydrocodeine/Promethazine |
| Aspirin/Caffeine/Hydrocodone |
| Aspirin/Caffeine/Propoxyphene |
| Aspirin/Carisoprodol/Codeine |
| Aspirin/Codeine |
| Aspirin/Hydrocodone |
| Aspirin/Oxycodone |
| Aspirin/Pentazocine |
| Aspirin/Propoxyphene |
| Buprenorphine |
| Buprenorphine/Naloxone |
| Codeine |
| Codeine/Guaifenesin |
| Codeine/Papaverine |
| Codeine/Promethazine |
| Codeine/Pseudoephedrine |
| Dihydrocodeine |
| Fentanyl |
| Homatropine/Hydrocodone |
| Hydrocodone |
| Hydrocodone/Ibuprofen |
| Hydrocodone/Phenyltoloxamine |
| Hydrocodone/Pseudoephedrine |
| Hydromorphone |
| Levorphanol |
| Meperidine |
| Meperidine/Promethazine |
| Methadone |
| Morphine |
| Morphine/Naltrexone |
| Naloxone/Pentazocine |
| Oxycodone |
| Oxycodone/Ibuprofen |
| Oxymorphone |
| Propoxyphene |
| Tapentadol |
| Tramadol |
| Tramadol/Acetaminophen |

**Supplemental Table 3. List of search terms for identifying prescriptions of medication for opioid use disorder**

| ‘%buprenorphine%nalox%’ | Buprenorphine with Naloxone as delivered as tablet (sublingual) or film (buccal) |
| --- | --- |
| ‘buprenorph%subling%’ | Buprenorphine alone or with Naloxone delivered as tablet or film (sublingual) |
| ‘%buprenorphine%implant%’ | Buprenorphine delivered as implant |
| ‘%buprenorphine%syringe%’ | Buprenorphine delivered by injection (syringe) |
| ‘%Methadone%’ (in form ‘%SOLN%’) | Methadone delivered orally as solution |

**Supplemental Table 4. Stop codes to identify intervention-related health care utilization in the Managerial Cost Accounting system for the Multidisciplinary Complex Pain Clinic (or similar standard of care in control group)**

| Stop Code | Stop Code Description As Labeled in Corporate Data Warehouse | Intervention-related utilization based on *OTGAR*^a^ *or other source* |
| --- | --- | --- |
| 159 | CIH TREATMENT | *OTGAR*^a^ |
| 177 | HBPC - OTHER | *OTGAR*^a^ |
| 195 | POLYTRMA TRNSIT REHAB IND | *OTGAR*^a^ |
| 196 | POLYTRMA TRNSIT REHAB GRP | *OTGAR*^a^ |
| 197 | POLYTRAUMA/TBI IND | *OTGAR*^a^ |
| 197 | WLA-SPEECH PATH POLYTRAUMA | *OTGAR*^a^ |
| 198 | POLYTRAUMA/TBI GRP | *OTGAR*^a^ |
| 198 | WLA-SPEECH PATH POLY GROUP | *OTGAR*^a^ |
| 199 | TELEPHONE POLYTRAUMA/TBI | *OTGAR*^a^ |
| 199 | WLA-SPEECH PATH POLY TELE | *OTGAR*^a^ |
| 201 | PHYSICAL MED & REHAB SVC | *OTGAR*^a^ |
| 201 | PM&RS PHYSICIAN | *OTGAR*^a^ |
| 202 | RECREATION THERAPY SERVICE | *OTGAR*^a^ |
| 205 | PHYSICAL THERAPY | *OTGAR*^a^ |
| 206 | OCCUPATIONAL THERAPY | *OTGAR*^a^ |
| 207 | PM&RS INCENTIVE THERAPY | *OTGAR*^a^ |
| 208 | PM&RS COMPENSATED WORK THERAPY | *OTGAR*^a^ |
| 208 | PMRS CWT/TWE FACE TO FACE | *OTGAR*^a^ |
| 210 | SPINAL CORD INJURY | *OTGAR*^a^ |
| 211 | PM&RS AMP CLINIC | *OTGAR*^a^ |
| 213 | PM&RS VOCATIONAL ASSISTANCE | *OTGAR*^a^ |
| 214 | KINESIOTHERAPY | *OTGAR*^a^ |
| 215 | SCI HOME CARE PROGRAM | *OTGAR*^a^ |
| 219 | TBI (TRAUMATIC BRAIN INJURY) | *OTGAR*^a^ |
| 222 | PMRS CWT/SE FACE TO FACE | *OTGAR*^a^ |
| 223 | PMRS CWT/SE NON-F TO F (MAS NO | *OTGAR*^a^ |
| 228 | PMRS CWT/TWE NON-F TO F (MAS N | *OTGAR*^a^ |
| 230 | PM&RS DRIVER TRAINING | *OTGAR*^a^ |
| 295 | OBSERVATION SPINAL CORD INJURY | *OTGAR*^a^ |
| 296 | OBSERVATION REHABILITATION | *OTGAR*^a^ |
| 372 | WEIGHT MGMT & MOVE! PROG - IND | *OTGAR*^a^ |
| 373 | WEIGHT MGMT & MOVE! PROG - GRP | *OTGAR*^a^ |
| 417 | PROSTHETICS/ORTHOTICS | *OTGAR*^a^ |
| 418 | AMPUTATION CLINIC | *OTGAR*^a^ |
| 420 | PAIN CLINIC | *OTGAR*^a^ |
| 436 | CHIROPRACTIC CARE | *OTGAR*^a^ |
| 513 | SUBSTANCE ABUSE - INDIVIDUAL | Used by mental health providers in MCPC^b^ |
| 513 | SUBSTANCE USE DISORDER IND | Used by mental health providers in MCPC^b^ |
| 527 | TELEPHONE MH | Used by mental health providers in MCPC^b^ |

^a^ OTGAR: Opioid Therapy Guideline Adherence Report; ^b^ MCPC: Multidisciplinary complex pain clinics

**Supplemental Table 5. Benzodiazepine search terms for identifying benzodiazepine prescriptions**

| ‘%ALPRAZOLAM% | Alprazolam |
| --- | --- |
| ‘%CHLORDIAZEPOXIDE% | Chlordiazepoxide |
| %CLONAZEPAM%' | Chlonazepam |
| %CLORAZEPATE%' | Clorazepate |
| '%DIAZEPAM%' | Diazepam |
| ‘%ESTAZOLAM%' | Estazolam |
| ‘%FLURAZEPAM%' | Flurazepam |
| '%LORAZEPAM%' | Lorazepam |
| '%OXAZEPAM%' | Oxazepam |
| %QUAZEPAM%' | Quazepam |
| '%TEMAZEPAM%' | Temazepam |
| '%TRIAZOLAM%' | Triazolam |

**Supplemental Table 6. International Classification of Diseases-10 diagnosis codes to define diagnoses and overdose**

| **Opioid Use Disorder** | F11.10; F11.11 ; F11.120; F11.121; F11.122; F11.129; F11.14; F11.150; F11.151; F11.159; F11.181; F11.182; F11.188; F11.19; F11.20; F11.21; F11.220; F11.221; F11.222; F11.229; F11.23; F11.24; F11.250; F11.251; F11.259; F11.281; F11.282; F11.288; F11.29; |
| --- | --- |
| **Stimulant Use Disorder** | F14.10; F14.11; F14.120; F14.121; F14.122; F14.129; F14.13; F14.14; F14.150; F14.151; F14.159; F14.180; F14.181; F14.182; F14.188; F14.19; F14.20; F14.21; F14.220; F14.221; F14.222; F14.229; F14.23; F14.24; F14.250; F14.251; F14.259; F14.280; F14.281; F14.282; F14.288; F14.29; F14.90; F14.920; F14.921; F14.922; F14.929; F14.93; F14.94; F14.950; F14.951; F14.959; F14.980; F14.981; F14.982; F14.988; F14.99; F15.10; F15.11; F15.120; F15.121; F15.122; F15.129; F15.13; F15.14; F15.150; F15.151; F15.159; F15.180; F15.181; F15.182; F15.188; F15.19; F15.20; F15.21; F15.220; F15.221; F15.222; F15.229; F15.23; F15.24; F15.250; F15.251; F15.259; F15.280; F15.281; F15.282; F15.288; F15.29; F15.90; F15.920; F15.921; F15.922; F15.929; F15.93; F15.94; F15.950; F15.951; F15.959; F15.980; F15.981; F15.982; F15.988; F15.99 |
| **Anxiety** | F06.4; F40.8; F40.9; F41.1; F41.3; F41.8; F41.9; F43.22; F43.23 |
| **Alcohol Use Disorder** | E24.4; F10.10; F10.11; F10.120; F10.121; F10.129; F10.14; F10.150; F10.151; F10.159; F10.180; F10.181; F10.182; F10.188; F10.19; F10.20; F10.21; F10.220; F10.221; F10.229; F10.230; F10.231; F10.232; F10.239; F10.24; F10.250; F10.251; F10.259; F10.26; F10.27; F10.280; F10.281; F10.282; F10.288; F10.29; F10.920; F10.921; F10.929; F10.94; F10.950; F10.951; F10.959; F10.96; F10.97; F10.980; F10.981; F10.982; F10.988; F10.99; G31.2; G62.1; G72.1; I42.6; K29.20; K29.21; K70.0; K70.10; K70.11; K70.2; K70.30; K70.31; K70.40; K70.41; K70.9; K85.2; K85.20; K85.21; K85.22; K86.0; O35.4XX0; O35.4XX1; O35.4XX2; O35.4XX3; O35.4XX4; O35.4XX5; O35.4XX9; O99.310; O99.311; O99.312; O99.313; O99.314; O99.315 |
| **Cannabis Use Disorder** | F12.10; F12.11; F12.120; F12.121; F12.122; F12.129; F12.150; F12.151; F12.159; F12.180; F12.188; F12.19; F12.20; F12.21; F12.220; F12.221; F12.222; F12.229; F12.23; F12.250; F12.251; F12.259; F12.280; F12.288; F12.29; F12.90; F12.920; F12.921; F12.922; F12.929; F12.93; F12.950; F12.951; F12.959; F12.980; F12.988; F12.99 |
| **Nicotine Dependence** | F17.200; F17.201; F17.203; F17.208; F17.209; F17.210; F17.211; F17.213; F17.218; F17.219; F17.220; F17.221; F17.223; F17.228; F17.229; F17.290; F17.291; F17.293; F17.298; F17.299 |
| **Bipolar Disorder** | F25.0; F30.10; F30.11; F30.12; F30.13; F30.2; F30.3; F30.4; F30.8; F30.9; F31.0; F31.10; F31.11; F31.12; F31.13; F31.2; F31.30; F31.31; F31.32; F31.4; F31.5; F31.60; F31.61; F31.62; F31.63; F31.64; F31.70; F31.71; F31.72; F31.73; F31.74; F31.75; F31.76; F31.77; F31.78; F31.81; F31.89; F31.9 |
| **Depression** | F06.31; F06.32; F32.0; F32.1; F32.2; F32.3; F32.4; F32.5; F32.8; F32.81; F32.89; F32.9; F33.0; F33.1; F33.2; F33.3; F33.40; F33.41; F33.42; F33.8; F33.9; F34.1; F34.8; F34.9; F43.21; F43.23 |
| **Post-Traumatic Stress Disorder** | F43.10; F43.11; F43.12 |
| **Other Mental Health Disorder** | F43.0; F43.20; F43.24; F43.25; F43.29; F43.21; F43.22; F43.23; F44.0; F44.1; F44.2; F44.4; F44.5; F44.6; F44.7; F44.81; F44.89; F44.9; F45.0; F45.1; F45.22; F45.8; F45.9; F48.1; F50.00; F50.01; F50.02; F50.2; F50.8; F50.81; F50.82; F50.89; F50.9; F63.0; F63.1; F63.2; F63.3; F63.81; F63.89; F63.9; F68.10; F68.11; F68.12; F68.13; F90.0; F90.1; F90.2; F90.8; F90.9 |
| **Opioid Overdose** | T40.0X1A; T40.0X1D; T40.0X1S; T40.0X2A; T40.0X2D; T40.0X2S; T40.0X3A; T40.0X3D; T40.0X3S; T40.0X4A; T40.0X4D; T40.0X4S; T40.0X5A; T40.0X5D; T40.0X5S; T40.1X1A; T40.1X1D; T40.1X1S; T40.1X2A; T40.1X2D; T40.1X2S; T40.1X3A; T40.1X3D; T40.1X3S; T40.1X4A; T40.1X4D; T40.1X4S; T40.2X1A; T40.2X1D; T40.2X1S; T40.2X2A; T40.2X2D; T40.2X2S; T40.2X3A; T40.2X3D; T40.2X3S; T40.2X4A; T40.2X4D; T40.2X4S; T40.2X5A; T40.2X5D; T40.2X5S; T40.3X1A; T40.3X1D; T40.3X1S; T40.3X2A; T40.3X2D; T40.3X2S; T40.3X3A; T40.3X3D; T40.3X3S; T40.3X4A; T40.3X4D; T40.3X4S; T40.3X5A; T40.3X5D; T40.3X5S; T40.4X1A; T40.4X1D; T40.4X1S; T40.4X2A; T40.4X2D; T40.4X2S; T40.4X3A; T40.4X3D; T40.4X3S; T40.4X4A; T40.4X4D; T40.4X4S; T40.4X5A; T40.4X5D; T40.4X5S; T40.601A; T40.601D; T40.601S; T40.602A; T40.602D; T40.602S; T40.603A; T40.603D; T40.603S; T40.604A; T40.604D; T40.604S; T40.605A; T40.605D; T40.605S; T40.691A; T40.691D; T40.691S; T40.692A; T40.692D; T40.692S; T40.693A; T40.693D; T40.693S; T40.694A; T40.694D; T40.694S; T40.695A; T40.695D; T40.695S |
| **Sedative Use Disorder** | F13.10; F13.11; F13.120; F13.121; F13.129; F13.14; F13.150; F13.151; F13.159; F13.180; F13.181; F13.182; F13.188; F13.19; F13.20; F13.21; F13.220; F13.221; F13.229; F13.230; F13.231; F13.232; F13.239; F13.24; F13.250; F13.251; F13.259; F13.26; F13.27; F13.280; F13.281; F13.282; F13.288; F13.29; F13.90; F13.920; F13.921; F13.929; F13.930; F13.931; F13.932; F13.939; F13.94; F13.950; F13.951; F13.959; F13.96; F13.97; F13.980; F13.981; F13.982; F13.988; F13.99 |
| **Other Substance Use Disorder (Hallucinogens, Psychoactive, Inhalant)** | ; F16.10; F16.11; F16.120; F16.121; F16.122; F16.129; F16.14; F16.150; F16.151; F16.159; F16.180; F16.183; F16.188; F16.19; F16.20; F16.21; F16.220; F16.221; F16.229; F16.24; F16.250; F16.251; F16.259; F16.280; F16.283; F16.288; F16.29; F16.90; F16.920; F16.921; F16.929; F16.94; F16.950; F16.951; F16.959; F16.980; F16.983; F16.988; F16.99; F18.10; F18.11; F18.120; F18.121; F18.129; F18.14; F18.150; F18.151; F18.159; F18.17; F18.180; F18.188; F18.19; F18.20; F18.21; F18.220; F18.221; F18.229; F18.24; F18.250; F18.251; F18.259; F18.27; F18.280; F18.288; F18.29; F18.90; F18.920; F18.921; F18.929; F18.94; F18.950; F18.951; F18.959; F18.97; F18.980; F18.988; F18.99; F19.10; F19.11; F19.120; F19.121; F19.122; F19.129; F19.14; F19.150; F19.151; F19.159; F19.16; F19.17; F19.180; F19.181; F19.182; F19.188; F19.19; F19.20; F19.21; F19.220; F19.221; F19.222; F19.229; F19.230; F19.231; F19.232; F19.239; F19.24; F19.250; F19.251; F19.259; F19.26; F19.27; F19.280; F19.281; F19.282; F19.288; F19.29; F19.90; F19.920; F19.921; F19.922; F19.929; F19.930; F19.931; F19.932; F19.939; F19.94; F19.950; F19.951; F19.959; F19.96; F19.97; F19.980; F19.981; F19.982; F19.988; F19.99 |

**Supplemental Table 7. Demographic and clinical characteristics of treated and control populations at each site including standardized mean differences (SMD) for each variable**

**Table 7a. Site 1**

|  | **Unweighted** | |  | **Weighted** | |  |
| --- | --- | --- | --- | --- | --- | --- |
|  | **Case (N)** | **Control (N)** | **SMD** | **Case (N)** | **Control (N)** | **SMD** |
| Total N | 322 | 2898 |  | 3206 | 3219 |  |
| Average Age | 59.50 | 62.31 | 0.237 | 61.06 | 62.05 | 0.083 |
| Race |  |  | 0.090 |  |  | 0.031 |
| White | 63% (202) | 60% (1752) |  | 61% (1956) | 61% (1952) |  |
| Black | 33% (106) | 35% (1008) |  | 35% (1118) | 35% (1115) |  |
| Other Race/Hispanic Ethnicity | 2% (6) | 3% (84) |  | 2% (77) | 3% (90) |  |
| Unknown Race/Ethnicity | 2% (8) | 2% (54) |  | 2% (54) | 2% (62) |  |
| Female | 9% (28) | 8% (234) | 0.022 | 9% (274) | 8% (262) | 0.014 |
| Diagnoses, Prescriptions, Inpatient Care |  |  |  |  |  |  |
| OUD^a^ or MOUD^b^ | 44% (143) | 28% (801) | 0.354 | 30% (951) | 29% (943) | 0.009 |
| Benzodiazepines | 16% (52) | 11% (330) | 0.138 | 13% (430) | 12% (382) | 0.046 |
| Alcohol Use Disorder | 25% (82) | 22% (627) | 0.090 | 23% (735) | 22% (709) | 0.021 |
| Stimulant Use Disorder | 15% (49) | 10% (284) | 0.164 | 11% (361) | 10% (333) | 0.030 |
| Sedative Use Disorder | 3% (10) | 2% (61) | 0.063 | 3% (88) | 2% (71) | 0.035 |
| Cannabis Use Disorder | 13% (43) | 9% (263) | 0.136 | 10% (331) | 9% (306) | 0.027 |
| Other Substance Use Disorder | 8% (26) | 7% (204) | 0.039 | 7% (240) | 7% (230) | 0.013 |
| Depression, Anxiety, and/or PTSD | 77% (248) | 66% (1925) | 0.237 | 70% (2236) | 68% (2173) | 0.048 |
| Opioid Overdose | 7% (22) | 2% (66) | 0.183 | 3% (106) | 3% (84) | 0.021 |
| Inpatient admit prior to enrollment | 22% (72) | 10% (294) | 0.335 | 11% (362) | 11% (365) | 0.002 |

^a^ OUD— Opioid use disorder

^b^ MOUD – Medication for opioid use disorder

**Table 7b. Site 2**

|  | **Unweighted** | |  | **Weighted** | |  |
| --- | --- | --- | --- | --- | --- | --- |
|  | **Case (N)** | **Control (N)** | **SMD** | **Case (N)** | **Control (N)** | **SMD** |
| Total N | 259 | 2331 |  | 2596 | 2590 |  |
| Average Age | 63.68 | 64.89 | 0.100 | 64.57 | 64.80 | 0.006 |
| Race |  |  | 0.298 |  |  | 0.020 |
| White | 84% (217) | 83% (1946) |  | 84% (2183) | 84% (2164) |  |
| Black | 2% (4) | 7% (160) |  | 6% (154) | 6% (164) |  |
| Other Race/Hispanic Ethnicity | 9% (24) | 6% (134) |  | 6% (160) | 6% (158) |  |
| Unknown Race/Ethnicity | 5% (14) | 4% (91) |  | 4% (99) | 4% (104) |  |
| Female | 9% (24) | 8% (192) | 0.036 | 9% (241) | 8% (216) | 0.034 |
| Diagnoses, Prescriptions, Inpatient Care |  |  |  |  |  |  |
| OUD^a^ or MOUD^b^ | 14% (37) | 17% (404) | 0.083 | 18% (481) | 17% (441) | 0.005 |
| Benzodiazepines | 25% (64) | 26% (596) | 0.020 | 25% (643) | 25% (660) | 0.005 |
| Alcohol Use Disorder | 15% (40) | 15% (341) | 0.023 | 15% (391) | 15% (381) | 0.019 |
| Stimulant Use Disorder | 3% (7) | 4% (103) | 0.093 | 4% (100) | 4% (110) | 0.032 |
| Sedative Use Disorder | 2% (5) | 3% (66) | 0.059 | 3% (73) | 3% (71) | 0.016 |
| Cannabis Use Disorder | 6% (16) | 6% (137) | 0.013 | 6% (160) | 6% (153) | 0.012 |
| Other Substance Use Disorder | 3% (8) | 3% (61) | 0.028 | 3% (71) | 3% (69) | 0.016 |
| Depression, Anxiety, and/or PTSD | 61% (158) | 63% (1475) | 0.047 | 64% (1662) | 63% (1633) | 0.042 |
| Opioid Overdose | 2% (6) | 2% (36) | 0.056 | 1% (39) | 2% (42) | 0.014 |
| Inpatient admit prior to enrollment | 12% (31) | 11% (248) | 0.042 | 10% (260) | 11% (279) | 0.039 |

^a^ OUD— Opioid use disorder

^b^ MOUD – Medication for opioid use disorder

**Table 7c. Site 3**

|  | **Unweighted** | |  | **Weighted** | |  |
| --- | --- | --- | --- | --- | --- | --- |
|  | **Case (N)** | **Control (N)** | **SMD** | **Case (N)** | **Control (N)** | **SMD** |
| Total N | 111 | 999 |  | 1114 | 1110 |  |
| Average Age | 66.20 | 62.70 | 0.275 | 63.52 | 62.95 | 0.046 |
| Race |  |  | 0.142 |  |  | 0.081 |
| White | 92% (102) | 89% (894) |  | 92% (1022) | 90% (996) |  |
| Black | 2% (2) | 4% (38) |  | 2% (27) | 4% (40) |  |
| Other Race/Hispanic Ethnicity | 4% (4) | 5% (47) |  | 4% (49) | 5% (51) |  |
| Unknown Race/Ethnicity | 3% (3) | 2% (20) |  | 2% (17) | 2% (23) |  |
| Female | 11% (12) | 6% (64) | 0.158 | 7% (74) | 7% (76) | 0.008 |
| Diagnoses, Prescriptions, Inpatient Care |  |  |  |  |  |  |
| OUD^a^ or MOUD^b^ | 53% (59) | 46% (463) | 0.136 | 41% (456) | 47% (521) | 0.122 |
| Benzodiazepines | 29% (32) | 24% (241) | 0.107 | 26% (293) | 25% (273) | 0.037 |
| Alcohol Use Disorder | 32% (35) | 33% (327) | 0.026 | 33% (373) | 33% (362) | 0.018 |
| Stimulant Use Disorder | 8% (9) | 17% (165) | 0.258 | 15% (171) | 16% (174) | 0.009 |
| Sedative Use Disorder | 5% (6) | 6% (64) | 0.042 | 6% (63) | 6% (70) | 0.029 |
| Cannabis Use Disorder | 13% (14) | 15% (150) | 0.070 | 18% (203) | 15% (164) | 0.092 |
| Other Substance Use Disorder | 8% (9) | 10% (95) | 0.049 | 8% (84) | 9% (104) | 0.064 |
| Depression, Anxiety, and/or PTSD | 77% (86) | 72% (720) | 0.124 | 72% (797) | 73% (806) | 0.025 |
| Opioid Overdose | 0% (0) | 3% (29) | 0.244 | 0% (0) | 3% (29) | 0.232 |
| Inpatient Admit prior to enrollment | 23% (25) | 31% (314) | 0.201 | 32% (351) | 31% (339) | 0.020 |

^a^ OUD— Opioid use disorder

^b^ MOUD – Medication for opioid use disorder

**Supplemental Table 8.** Results of intervention and total downstream cost event studies and baseline/three-month averages

|  |  |  | **Mean Costs (Standard Error)^c^** | |
| --- | --- | --- | --- | --- |
|  | **Pre-test  p-value^a^** | **Average Treatment Effect of the Treated^b^** | **Baseline** | **Three months after  clinic opened** |
| **Intervention Costs** | | | | |
| **Site 1** | 0.055 | $220.93 (95%CI 138.17, 303.70) p<0.001 | Treated: $438 (47)  Control: $298 (13) | Treated: $630 (57)  Control: $414 (21) |
| **Site 2** | 0.398 | $271.05 (95%CI 145.46, 396.64) p<0.001 | Treated: $371 (58)  Control: $484 (30) | Treated: $416 (54)  Control: $501 (28) |
| **Site 3** | 0.037 | $253.93 (95%CI -24.33, 532.18) p=0.069 | Treated: $754 (190)  Control: $881 (64) | Treated: $815 (136)  Control: $1118 (68) |
| **Downstream Costs** | | | | |
| **Site 1** | 0.438 | $1555.93 (95%CI 492.13, 2619.73) p<0.005 | Treated: $6,773 (834)  Control: $7,042 (376) | Treated: $11,851 (1664)  Control: $8,632 (404) |
| **Site 2** | 0.289 | $1217.93 (95%CI -64.62, 2500.48) p=0.054 | Treated: $7848 (1275)  Control: $9751 (597) | Treated: $10,460 (1625)  Control: $11,127 (634) |
| **Site 3** | 0.470 | $3791.27 (95%CI -690.46, 8273.01) p=0.092 | Treated: $15,413 (3158)  Control: $13,736 (929) | Treated: $23,863 (7295)  Control: $16,372 (1027) |

^a^ Difference-in-difference event study pre-test results for parallel trends using the Callaway Sant’anna framework with propensity score weights

^b^ Overall Average Treatment Effect of the Treated (ATT) for the difference-in-difference event studies using the Callaway Sant’anna framework with propensity score weights

^c^ Average intervention or downstream costs for control and treated groups at baseline and 3-months after the opening of the clinic at each site

**Supplemental Table 9a-c: Propensity-score weighted difference-in-difference event study estimates for site-level intervention costs using the Callaway-Sant’anna framework**

**Supplemental Table 9a. Site 1**

| **Time Interval** | **Estimate** | **Std. Error** | **95% Confidence Intervals** |
| --- | --- | --- | --- |
| Nov-Jan 2019 | 17.61 | 31.77 | (-74.39, 109.61) |
| Feb-Apr 2019 | -17.71 | 30.74 | (-106.75,71.33) |
| May-Jul 2019 | 22.84 | 26.71 | (-54.52,100.21) |
| Aug-Oct 2019 | -40.48 | 37.50 | (-149.09,68.13) |
| Nov-Jan 2020 | 26.70 | 44.57 | (-102.4,155.8) |
| Feb-Apr 2020 | 38.81 | 50.99 | (-108.86,186.48) |
| May-Jul 2020 | 103.16 | 47.96 | (-35.74,242.06) |
| Aug-Oct 2020 | 68.87 | 46.42 | (-65.56,203.3) |
| Nov-Jan 2021 | 350.36 | 64.35 | (163.99,536.73) |
| Feb-Apr 2021 | 327.60 | 73.53 | (114.63,540.58) |
| May-Jul 2021 | 122.22 | 65.34 | (-67.01,311.46) |
| Aug-Oct 2021 | 306.90 | 70.96 | (101.39,512.4) |
| Nov-Jan 2022 | 252.60 | 83.22 | (11.58,493.63) |
| Feb-Apr 2022 | 188.03 | 60.49 | (12.85,363.21) |
| May-Jul 2022 | 150.87 | 61.50 | (-27.24,328.97) |

**Supplemental Table 9b. Site 2**

| **Time Interval** | **Estimate** | **Std. Error** | **95% Confidence Interval** |
| --- | --- | --- | --- |
| May-Jul 2019 | -1.35 | 28.59 | (-80.79, 78.09) |
| Aug-Oct 2019 | -16.55 | 34.54 | (-112.52, 79.42) |
| Nov-Jan 2020 | -0.18 | 47.1 | (-131.05, 130.68) |
| Feb-Apr 2020 | 126.83 | 55.21 | (-26.58, 280.24) |
| May-Jul 2020 | -41.47 | 50.96 | (-183.08, 100.14) |
| Aug-Oct 2020 | -17.99 | 50.02 | (-156.99, 121.01) |
| Nov-Jan 2021 | 27.76 | 70.61 | (-168.46, 223.97) |
| Feb-Apr 2021 | 14.25 | 61.83 | (-157.57, 186.06) |
| May-Jul 2021 | 27.94 | 65.04 | (-152.79, 208.67) |
| Aug-Oct 2021 | 285.81 | 104.84 | (-5.51, 577.13) |
| Nov-Jan 2022 | 421.8 | 118.9 | (91.41, 752.19) |
| Feb-Apr 2022 | 368.86 | 99.79 | (91.57, 646.14) |
| May-Jul 2022 | 307.12 | 82.16 | (78.83, 535.41) |
| Aug-Oct 2022 | 441.25 | 104.04 | (152.14, 730.36) |
| Nov-Jan 2023 | 301.38 | 92.59 | (44.09, 558.67) |

**Supplemental Table 9c. Site 3**

| **Time Interval** | **Estimate** | | **Std. Error** | **95% Confidence Interval** |
| --- | --- | --- | --- | --- |
| Jun-Aug 2019 | | 221.53 | 130.23 | (-124.84, 567.89) |
| Sep-Nov 2019 | | -46.17 | 163.77 | (-481.76, 389.42) |
| Dec-Feb 2020 | | -311.65 | 115.01 | (-617.54, -5.76) |
| Mar-May 2020 | | 152.8 | 158.11 | (-267.72, 573.33) |
| Jun-Aug 2020 | | -73.54 | 78.7 | (-282.86, 135.77) |
| Sep-Nov 2020 | | 24.07 | 114.41 | (-280.23, 328.37) |
| Dec-Feb 2021 | | 75.47 | 155.27 | (-337.5, 488.44) |
| Mar-May 2021 | | 89.44 | 187.21 | (-408.48, 587.36) |
| Jun-Aug 2021 | | 330.38 | 199.73 | (-200.86, 861.61) |
| Sep-Nov 2021 | | 203.72 | 170.87 | (-250.74, 658.19) |
| Dec-Feb 2022 | | 166.26 | 156.14 | (-249.03, 581.54) |
| Mar-May 2022 | | 501.06 | 237.33 | (-130.18, 1132.29) |
| Jun-Aug 2022 | | 289.86 | 171.2 | (-165.48, 745.19) |
| Sep-Nov 2022 | | 282.67 | 189.09 | (-220.24, 785.59) |
| Dec-Feb 2023 | | 168.05 | 175.22 | (-297.98, 634.08) |

**Supplemental Table 10a-c: Propensity-score weighted difference-in-difference event study estimates for site-level total downstream costs using the Callaway-Sant’anna framework**

**Supplemental Table 10a. Site 1**

| **Time Interval** | **Estimate** | **Std. Error** | **95% Confidence Interval** |
| --- | --- | --- | --- |
| Nov-Jan 2019 | 904.59 | 646.24 | (-884.25, 2693.43) |
| Feb-Apr 2019 | -772.7 | 653.23 | (-2580.89, 1035.49) |
| May-Jul 2019 | 489.33 | 614.82 | (-1212.53, 2191.19) |
| Aug-Oct 2019 | -417.17 | 658.61 | (-2240.23, 1405.9) |
| Nov-Jan 2020 | 428.72 | 630.88 | (-1317.59, 2175.03) |
| Feb-Apr 2020 | 1413.7 | 953.74 | (-1226.31, 4053.71) |
| May-Jul 2020 | -1367.65 | 870.28 | (-3776.64, 1041.35) |
| Aug-Oct 2020 | 930.24 | 727.87 | (-1084.55, 2945.03) |
| Nov-Jan 2021 | 1363.81 | 755.36 | (-727.08, 3454.7) |
| Feb-Apr 2021 | 526.09 | 706.12 | (-1428.49, 2480.67) |
| May-Jul 2021 | 911.59 | 845.92 | (-1429.98, 3253.16) |
| Aug-Oct 2021 | 2435.18 | 931.21 | (-142.47, 5012.82) |
| Nov-Jan 2022 | 2005.67 | 894.03 | (-469.07, 4480.41) |
| Feb-Apr 2022 | 2767.21 | 890.5 | (302.24, 5232.17) |
| May-Jul 2022 | 1507.66 | 930.69 | (-1068.56, 4083.87) |

**Supplemental Table 10b. Site 2**

| **Time Interval** | **Estimate** | **Std. Error** | **95% Confidence Interval** |
| --- | --- | --- | --- |
| May-Jul 2019 | 136.36 | 834.53 | (-2159.94, 2432.66) |
| Aug-Oct 2019 | 1201.76 | 1144.09 | (-1946.33, 4349.86) |
| Nov-Jan 2020 | 89.97 | 1198.21 | (-3207.04, 3386.97) |
| Feb-Apr 2020 | -1065.89 | 841.79 | (-3382.17, 1250.39) |
| May-Jul 2020 | -351.06 | 856.25 | (-2707.12, 2005.01) |
| Aug-Oct 2020 | -255.45 | 784.19 | (-2413.24, 1902.35) |
| Nov-Jan 2021 | -71.36 | 768.44 | (-2185.8, 2043.08) |
| Feb-Apr 2021 | 1039.72 | 835.55 | (-1259.39, 3338.82) |
| May-Jul 2021 | 469.02 | 920.48 | (-2063.77, 3001.8) |
| Aug-Oct 2021 | -51.15 | 884.51 | (-2484.97, 2382.67) |
| Nov-Jan 2022 | 1496.53 | 957.16 | (-1137.18, 4130.25) |
| Feb-Apr 2022 | 2388.72 | 1075.73 | (-571.26, 5348.7) |
| May-Jul 2022 | 1668.85 | 820.4 | (-588.56, 3926.26) |
| Aug-Oct 2022 | 931.4 | 738.22 | (-1099.88, 2962.68) |
| Nov-Jan 2023 | 1800.36 | 810.76 | (-430.54, 4031.25) |

**Supplemental 10c. Site 3**

| **Time Interval** | **Estimate** | **Std. Error** | **95% Confidence Interval** |
| --- | --- | --- | --- |
| Jun-Aug 2019 | -1284.01 | 1879.15 | (-6198.29, 3630.27) |
| Sep-Nov 2019 | -1584.95 | 3207.42 | (-9972.88, 6802.98) |
| Dec-Feb 2020 | -112.81 | 1690.62 | (-4534.06, 4308.45) |
| Mar-May 2020 | 5376.61 | 4475.87 | (-6328.53, 17081.75) |
| Jun-Aug 2020 | 136.32 | 2487.68 | (-6369.38, 6642.02) |
| Sep-Nov 2020 | 186.77 | 3928.97 | (-10088.13, 10461.66) |
| Dec-Feb 2021 | -3562.79 | 2748.43 | (-10750.37, 3624.79) |
| Mar-May 2021 | 3299.82 | 3790.53 | (-6613.03, 13212.66) |
| Jun-Aug 2021 | 2196.34 | 3053.42 | (-5788.84, 10181.52) |
| Sep-Nov 2021 | 4549.68 | 6791.16 | (-13210.31, 22309.67) |
| Dec-Feb 2022 | 1031.04 | 2388.04 | (-5214.08, 7276.16) |
| Mar-May 2022 | 1655.83 | 2469.38 | (-4802, 8113.66) |
| Jun-Aug 2022 | 5325.83 | 4777.25 | (-7167.45, 17819.11) |
| Sep-Nov 2022 | 6722.24 | 3629.92 | (-2770.61, 16215.08) |
| Dec-Feb 2023 | 5549.42 | 3476.74 | (-3542.82, 14641.67) |
