# Supplementary material for: Implementation, intervention, and downstream costs for implementation of a multidisciplinary complex pain clinic in the Veterans Health Administration
Source: Health Serv Res. 2024 Jul 2;59(Suppl 2):e14345. doi: 10.1111/1475-6773.14345 (PMC11540574; doi:10.1111/1475-6773.14345)

Supplemental Figure 3a-c: Propensity-score weighted two-way fixed effects event studies for site-level intervention costs

Supplemental Figure 3a. Site 1.

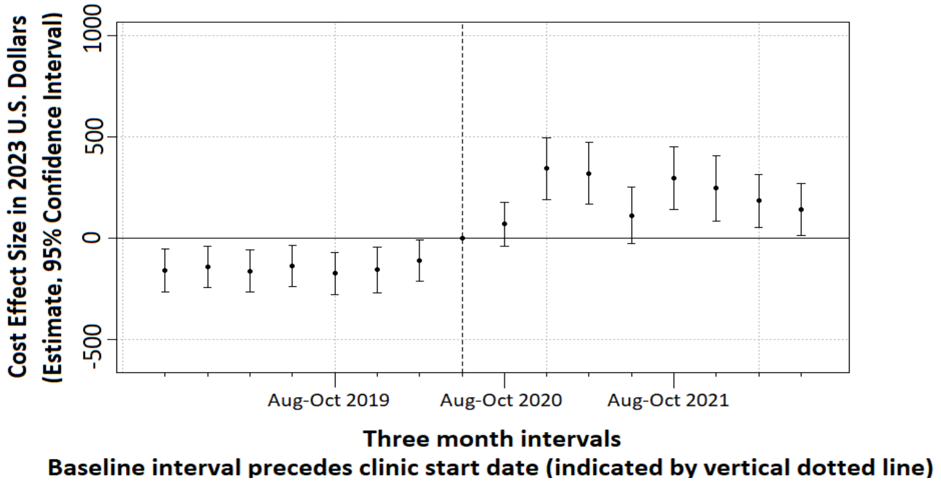

Supplemental Figure 3b. Site 2.

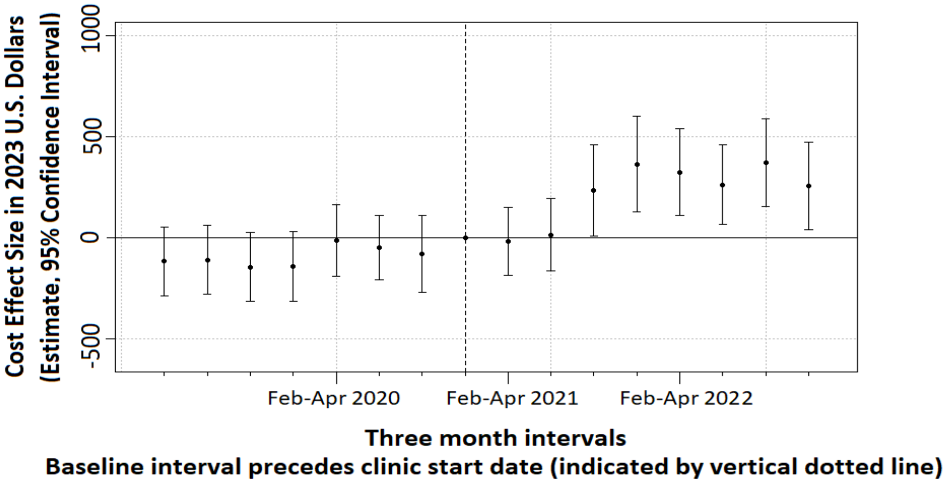

Supplemental Figure 3c. Site 3

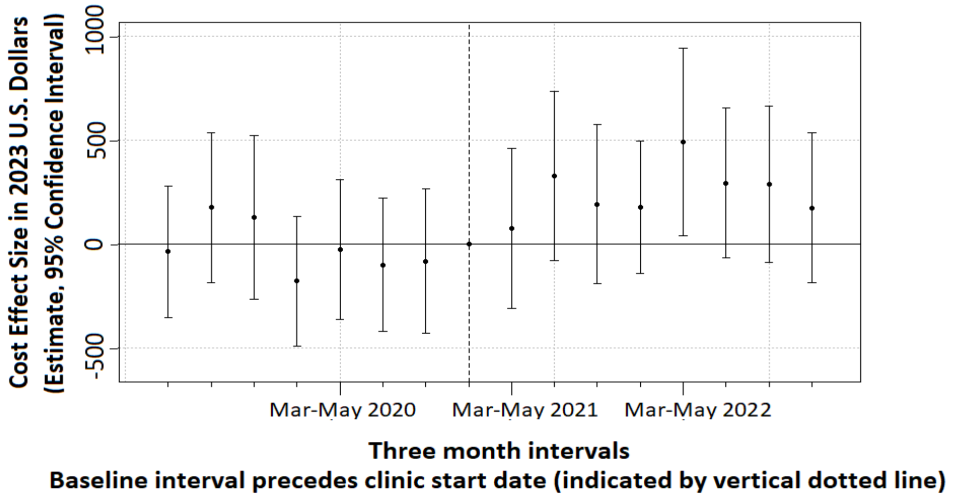

Supplement: Supplementary file 4 — Supplemental Figure 3a‐c: Propensity‐score weighted two‐way fixed effects event studies for site‐level intervention costs [file HESR-59-0-s006.pdf]
