# Supplementary material for: Implementation, intervention, and downstream costs for implementation of a multidisciplinary complex pain clinic in the Veterans Health Administration
Source: Health Serv Res. 2024 Jul 2;59(Suppl 2):e14345. doi: 10.1111/1475-6773.14345 (PMC11540574; doi:10.1111/1475-6773.14345)

Supplemental Figure 4a-c: Propensity-score weighted two-way fixed effects event studies for site-level total downstream costs

Supplemental Figure 4a. Site 1

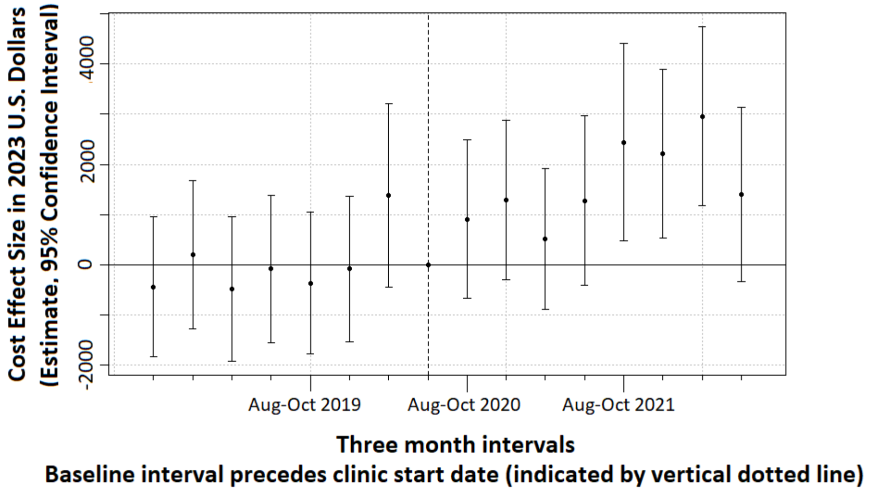

Supplemental Figure 4b. Site 2

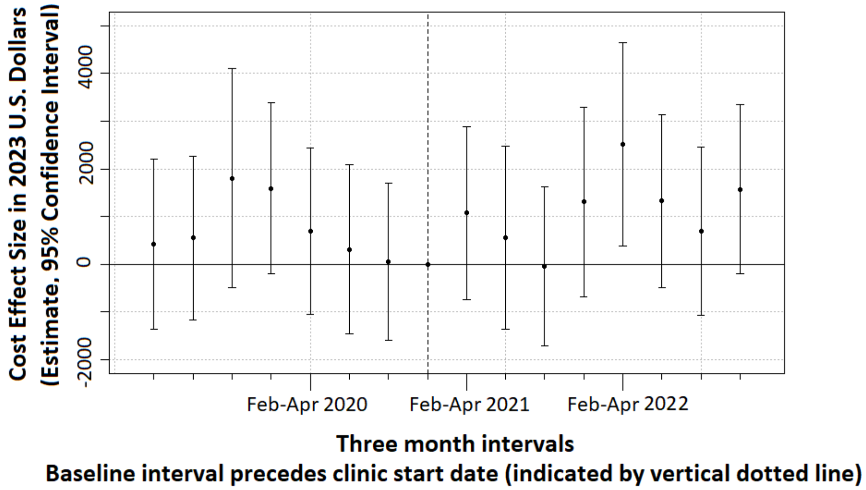

Supplemental Figure 4c. Site 3

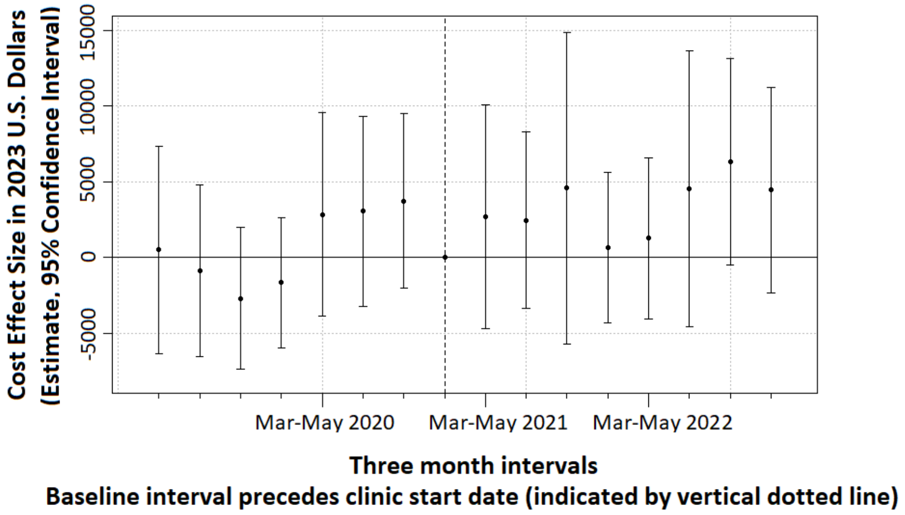

Supplement: Supplementary file 5 — Supplemental Figure 4a‐c: Propensity‐score weighted two‐way fixed effects event studies for site‐level total downstream costs. [file HESR-59-0-s004.pdf]
