# Supplementary material for: Implementation, intervention, and downstream costs for implementation of a multidisciplinary complex pain clinic in the Veterans Health Administration
Source: Health Serv Res. 2024 Jul 2;59(Suppl 2):e14345. doi: 10.1111/1475-6773.14345 (PMC11540574; doi:10.1111/1475-6773.14345)

Supplemental Figure 5a-c: Propensity-score weighted difference-in-difference event studies for site-level outpatient-only downstream costs

Supplemental Figure 5a. Site 1

Overall Average Treatment Effect of Treated (ATT): 842.14 (95% CI 227.44, 1456.83)   p<0.007

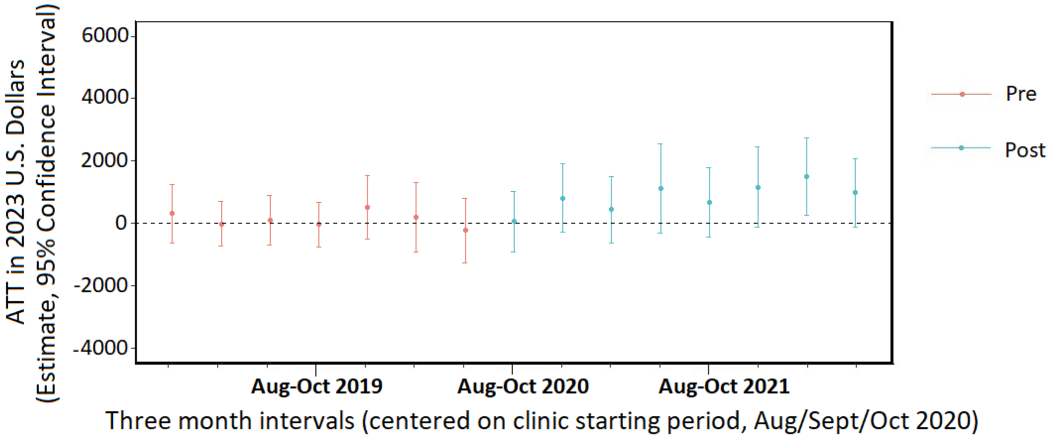

Supplemental Figure 5b. Site 2

Overall Average Treatment Effect of Treated (ATT): $891.08 (95% CI 114.17, 1667.99) p<0.015

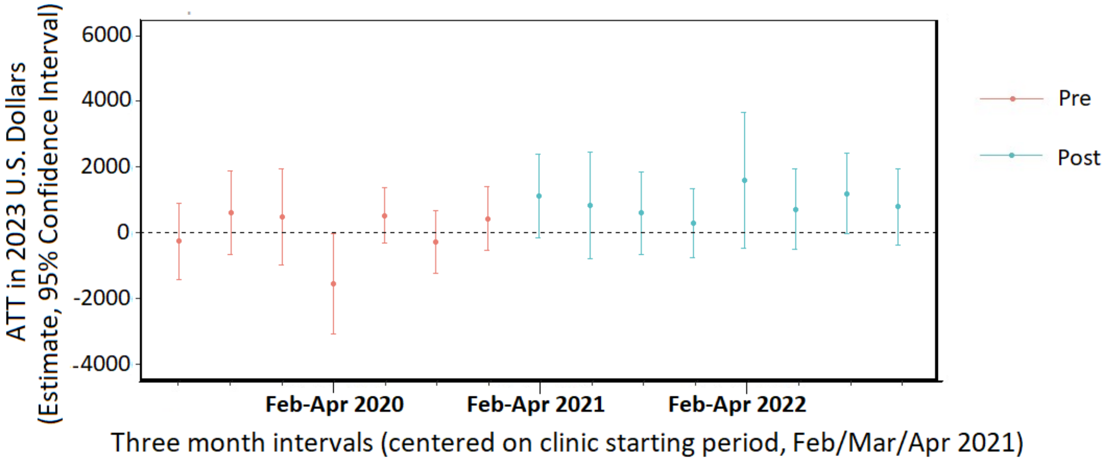

Supplemental Figure 5c. Site 3

Overall Average Treatment Effect of Treated (ATT): 2130. 51 (95% CI 688.92, 3592.10)   p<0.004

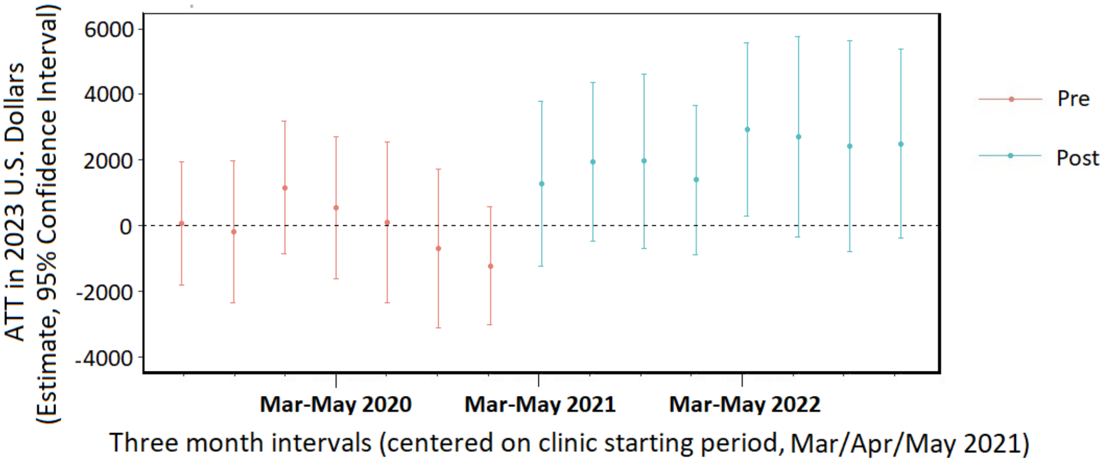

Supplement: Supplementary file 6 — Supplemental Figure 5a‐c: Propensity‐score weighted difference‐in‐difference event studies for site‐level outpatient‐only downstream costs. [file HESR-59-0-s001.pdf]
